# Representation learning for multi-modal spatially resolved transcriptomics data

**DOI:** 10.1093/bioinformatics/btag316

**Published:** 2026-05-21

**Authors:** Kalin Nonchev, Sonali Andani, Joanna Ficek-Pascual, Marta Nowak, Bettina Sobottka, Tumor Profiler Consortium, Rudolf Aebersold, Melike Ak, Faisal S Al-Quaddoomi, Silvana I Albert, Jonas Albinus, Ilaria Alborelli, Sonali Andani, Per-Olof Attinger, Marina Bacac, Daniel Baumhoer, Beatrice Beck-Schimmer, Niko Beerenwinkel, Christian Beisel, Lara Bernasconi, Anne Bertolini, Bernd Bodenmiller, Ximena Bonilla, Lars Bosshard, Byron Calgua, Ruben Casanova, Stéphane Chevrier, Natalia Chicherova, Ricardo Coelho, Maya D’Costa, Esther Danenberg, Natalie R Davidson, Monica-Andreea Dragan, Reinhard Dummer, Stefanie Engler, Martin Erkens, Katja Eschbach, Cinzia Esposito, André Fedier, Pedro F Ferreira, Joanna Ficek-Pascual, Anja L Frei, Bruno Frey, Sandra Goetze, Linda Grob, Gabriele Gut, Detlef Günther, Pirmin Haeuptle, Viola Heinzelmann-Schwarz, Sylvia Herter, Rene Holtackers, Tamara Huesser, Alexander Immer, Anja Irmisch, Francis Jacob, Andrea Jacobs, Tim M Jaeger, Katharina Jahn, Alva R James, Philip M Jermann, André Kahles, Abdullah Kahraman, Viktor H Koelzer, Werner Kuebler, Jack Kuipers, Christian P Kunze, Christian Kurzeder, Kjong-Van Lehmann, Mitchell Levesque, Ulrike Lischetti, Flavio C Lombardo, Sebastian Lugert, Gerd Maass, Markus G Manz, Philipp Markolin, Martin Mehnert, Julien Mena, Julian M Metzler, Nicola Miglino, Emanuela S Milani, Holger Moch, Simone Muenst, Riccardo Murri, Charlotte K Y Ng, Stefan Nicolet, Marta Nowak, Monica Nunez Lopez, Patrick G A Pedrioli, Lucas Pelkmans, Salvatore Piscuoglio, Michael Prummer, Laurie Prélot, Natalie Rimmer, Mathilde Ritter, Christian Rommel, María L Rosano-González, Gunnar Rätsch, Natascha Santacroce, Jacobo Sarabia del Castillo, Ramona Schlenker, Petra C Schwalie, Severin Schwan, Tobias Schär, Gabriela Senti, Wenguang Shao, Franziska Singer, Sujana Sivapatham, Berend Snijder, Bettina Sobottka, Vipin T Sreedharan, Stefan Stark, Daniel J Stekhoven, Tanmay Tanna, Alexandre P A Theocharides, Tinu M Thomas, Markus Tolnay, Vinko Tosevski, Nora C Toussaint, Mustafa A Tuncel, Marina Tusup, Audrey Van Drogen, Marcus Vetter, Tatjana Vlajnic, Sandra Weber, Walter P Weber, Rebekka Wegmann, Michael Weller, Fabian Wendt, Norbert Wey, Andreas Wicki, Mattheus H E Wildschut, Bernd Wollscheid, Shuqing Yu, Johanna Ziegler, Marc Zimmermann, Martin Zoche, Gregor Zuend, Viktor H. Koelzer, Gunnar Rätsch

**Affiliations:** Department of Computer Science, ETH Zurich, Universitätstrasse 6, Zurich 8092, Switzerland; Swiss Institute of Bioinformatics, Amphipôle, Quartier UNIL-Sorge, Zurich 1015, Switzerland; Department of Computer Science, ETH Zurich, Universitätstrasse 6, Zurich 8092, Switzerland; Swiss Institute of Bioinformatics, Amphipôle, Quartier UNIL-Sorge, Zurich 1015, Switzerland; Computational and Translational Pathology Group, Department of Pathology and Molecular Pathology, University Hospital Zurich, University of Zürich, Schmelzbergstrasse 12, Zurich 8091, Switzerland; Department of Computer Science, ETH Zurich, Universitätstrasse 6, Zurich 8092, Switzerland; Swiss Institute of Bioinformatics, Amphipôle, Quartier UNIL-Sorge, Zurich 1015, Switzerland; Computational and Translational Pathology Group, Department of Pathology and Molecular Pathology, University Hospital Zurich, University of Zürich, Schmelzbergstrasse 12, Zurich 8091, Switzerland; Computational and Translational Pathology Group, Department of Pathology and Molecular Pathology, University Hospital Zurich, University of Zürich, Schmelzbergstrasse 12, Zurich 8091, Switzerland; Computational and Translational Pathology Group, Department of Pathology and Molecular Pathology, University Hospital Zurich, University of Zürich, Schmelzbergstrasse 12, Zurich 8091, Switzerland; Department of Oncology, University of Oxford, Roosevelt Drive, Oxford OX3 7DQ, United Kingdom; Institute of Medical Genetics and Pathology, University Hospital Basel, Schönbeinstrasse 40, Basel 4031, Switzerland; Department of Computer Science, ETH Zurich, Universitätstrasse 6, Zurich 8092, Switzerland; Swiss Institute of Bioinformatics, Amphipôle, Quartier UNIL-Sorge, Zurich 1015, Switzerland; AI Center, ETH Zurich, Andreasstrasse 5, Zurich 8092, Switzerland; Medical Informatics Unit, University Hospital Zurich, Zurich 8091, Switzerland

## Abstract

**Motivation:**

Spatial transcriptomics enables in-depth molecular characterization of samples on a morphology and RNA level while preserving spatial location. Integrating the resulting multi-modal data is an unsolved problem, and developing new solutions in precision medicine depends on improved methodologies.

**Results:**

We introduce AESTETIK, a convolutional deep learning model that jointly integrates spatial, transcriptomics, and morphology information to learn accurate spot representations. AESTETIK yielded substantially improved cluster assignments on widely adopted technology platforms (e.g. 10x Genomics™, NanoString™) across multiple datasets. We achieved performance enhancement on structured tissues (e.g. brain) with a 21% increase in median ARI over previous state-of-the-art methods. Notably, AESTETIK also demonstrated superior performance on cancer tissues with heterogeneous cell populations, showing a 2-fold increase in breast cancer, 79% in melanoma, and 21% in liver cancer. We expect that these advances will enable a multi-modal understanding of key biological processes.

**Availability and implementation:**

AESTETIK is implemented in Python 3 and is available as open source software at http://www.github.com/ratschlab/aestetik. The Snakemake pipeline for reproducing the results is available at http://www.github.com/ratschlab/st-rep.

## 1 Introduction

**Figure 1:**
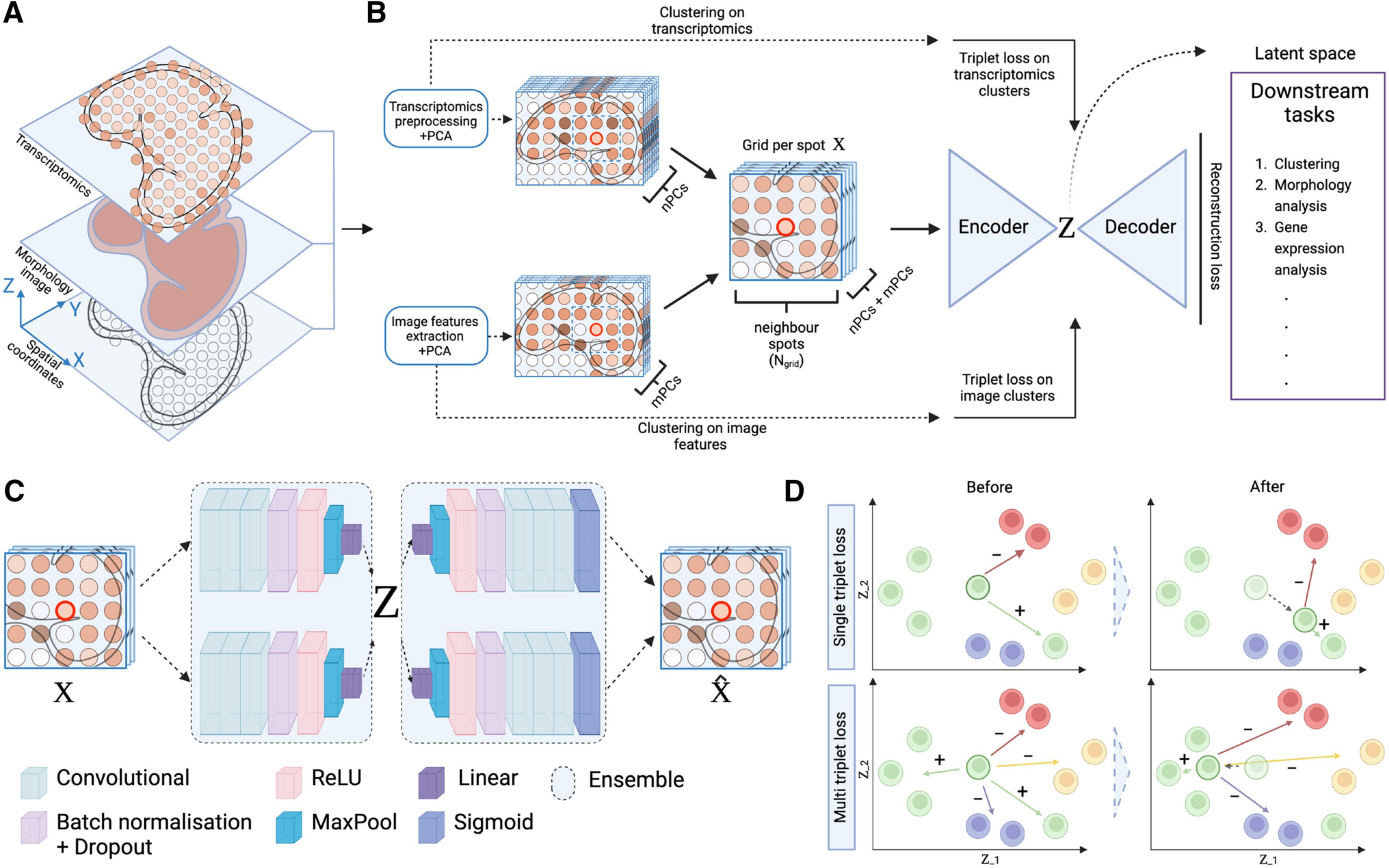
AESTETIK integrates spatial, transcriptomics, and morphology information to learn accurate spot representations. (A) Spatial transcriptomics enables in-depth molecular characterization of samples on a morphology and RNA level while preserving spatial location. (B) Workflow of AESTETIK. Initially, the transcriptomics and morphology spot representations are preprocessed. Next, a dimensionality reduction technique (e.g. PCA) is applied. Subsequently, the processed spot representations are clustered separately to acquire labels required for the multi-triplet loss. Afterwards, the modality-specific representations are fused through concatenation and the grid per spot is built. This is used as an input for the autoencoder. Lastly, the spatial-, transcriptomics-, and morphology-informed spot representations are obtained and used for downstream tasks such as clustering, morphology analysis, etc. (C) AESTETIK relies on a convolutional encoder–decoder architecture to learn accurate spot representations from the spatial transcriptomics data. (D) Using a multi-triplet loss, instead of a single triplet loss adds extra positive and negative instances per class around the anchor point, improving the placement of the anchor in the latent space.

In multicellular organisms, cells are organized into tissues, groups of cells exhibiting common characteristics related to the biological function (Asp *et al.* 2020, Rao *et al.* 2021). Recent advances in spatial transcriptomics enable in-depth molecular characterization of samples, capturing their morphology and RNA composition while retaining the spatial location (Fig. 1A). The gene expression profiles are usually available per spot, e.g. a 55 μm tissue region (10x Genomics™, Visium) covering the whole transcriptome (Williams *et al.* 2022), or at a single-cell resolution but with a limited number of captured genes (CosMx NanoString™). More recent spatial transcriptomics technologies provide whole-transcriptome coverage along with higher resolution (e.g. 2 μm, 10x Genomics™, Visium HD). Spatially aligning cell types by molecular phenotypes and morphology is important for understanding tissue-specific properties (e.g. neural organization in the brain, Lein *et al.* 2017) in a physiological state and in the context of disease progression and treatment (Ståhl *et al.* 2016, Chen *et al.* 2020, Yoosuf *et al.* 2020, Longo *et al.* 2021). Nevertheless, spatial transcriptomics analysis demands manual annotation of multi-modal data, representing a laborious and resource-intensive process. Achieving reliable automation and overcoming limitations in cross-modal expertise will lead to more accurate annotations, offering a comprehensive, multi-modal perspective on biological mechanisms and interactions (Zeng *et al.* 2022).

Despite recent progress, computational data analysis that integrates all available data modalities, i.e. spatial information, transcriptomics, and morphology, remains challenging. Most existing methods either fall short in effectively integrating all modalities, especially those adapted from single-cell analysis or are computationally expensive (Zeng *et al.* 2022). For example, *BayesSpace* uses a Bayesian approach with a prior giving higher weight to physically close spots (Zhao *et al.* 2021); *MUSE* relies on a multi-view autoencoder to learn a latent space from transcriptomics and morphology (Bao *et al.* 2022); *stLearn* quantifies morphological distance through histology image features and incorporates these distances with spatial neighbors to refine gene expression (Pham *et al.* 2023). Furthermore, alternative methods suggested a different perspective on modeling the spatial transcriptomics data by using graph neural networks (GNN) (Hu *et al.* 2021, Dong and Zhang 2022, Ren *et al.* 2022, Long *et al.* 2023), including *PearlST* (Wang *et al.* 2024), which integrates PDE-based gene expression augmentation with Wasserstein adversarial regularized graph learning. However, the expression profiles often suffer from biological variability (e.g. cell-cycle stage) (Eldar and Elowitz 2010, Kharchenko *et al.* 2014) or technical noise (Kharchenko *et al.* 2014, Buettner *et al.* 2015). GNNs’ inherent susceptibility to noise can undermine their robustness and performance in downstream applications (Dai *et al.* 2018, Zügner *et al.* 2018). Therefore, a new and reliable integration approach is needed to overcome the aforementioned challenges and improve spatial transcriptomics analysis at both spot and single-cell resolution, ensuring adaptability across spatial transcriptomics technologies.

To this end, we developed AESTETIK, a model that jointly integrates spatial, transcriptomics, and morphology information to learn accurate spot representations. We compared its performance against previous state-of-the-art methods on multiple datasets and widely adopted spatial transcriptomics platforms at both spot and single-cell resolution. Spot-level evaluations were conducted on brain tissue (Maynard *et al.* 2021), breast cancer (Wu *et al.* 2021), and newly generated, previously unreleased metastatic melanoma samples sequenced using Visium (10x Genomics™). Single-cell–resolution analyses were performed on liver samples from normal and cancer patients using CosMx (NanoString™). To further demonstrate the framework’s generalization to platforms without histological images, we additionally validated on a MERFISH mouse hypothalamus dataset (Moffitt *et al.* 2018). We substantially improved the clustering accuracy across all datasets which yielded spatial domains with coherent expression and morphology. Through an in-depth ablation study, we showed the enhanced value of utilizing all available data modalities given the specifics of the analyzed tissue, highlighting the importance of modality-aware model design. Further, we validated the learned representation by identifying the main biological drivers and characterizing clusters based on morphology and cell-type composition.

## 2 Materials and methods

### 2.1 Data preprocessing

AESTETIK takes in spatial, transcriptomics, and morphology information. We apply the same preprocessing pipeline across datasets and sequencing technologies. For simplicity, we refer to both spot and cell as a spot *(a single spot can contain 1 cell)*.

#### 2.1.1 Transcriptomics modality

Starting with raw counts, genes expressed in fewer than 10 spots are removed. Then, the *scanpy* function *highly variable genes* computes normalized variance in *Seurat v3* style, removing genes with variance below 1 (Lun *et al.* 2016, Wolf *et al.* 2018). Each spot undergoes normalization by total counts over all genes, followed by log1p transformation and scaling. Subsequently, PCA is applied to the preprocessed counts, extracting the first 15 PCs (Zhao *et al.* 2021).

#### 2.1.2 Morphology modality

The raw RGB image for each tissue slice is divided into tiles, each representing a spot and its defined neighborhood based on the spot diameter. Following the default preprocessing steps of *Inception v3* (Szegedy *et al.* 2016), the tiles are resized to 299×299 pixels, their center is cropped and the RGB channels are normalized. Morphology features are then extracted from the last network layer (with 2048 dimensions) of the pre-trained on *ImageNet* (Deng *et al.* 2009) deep-learning model *Inception v3*. Finally, PCA reduces the feature dimension from 2048 to 15.

#### 2.1.3 Grid construction

To begin, each spot is represented by two vectors containing the first $n_{d1}$ and $n_{d2}$ PCs obtained via PCA from the preprocessed transcriptomics and morphology modalities, along with their spatial coordinates. For simplicity, we assume $n_{d1}=n_{d2}=n_{\mathrm{pca}}$, but the following workflow holds also for $n_{d1}\neq n_{d2}$. These vectors are concatenated and scaled in the range [0,1]. Then, a square grid for each spot is constructed with the number of spatial neighbors, $N_{\mathrm{grid}}$, chosen as an odd number to ensure the center position of the selected spot in the window. This results in a tensor of size:


(1) $${\text{spot}}_{i}=(N_{\mathrm{grid}},N_{\mathrm{grid}},2*n_{\mathrm{pca}})$$

which can be interpreted as $N_{\mathrm{grid}}\times N_{\mathrm{grid}}$ image with $2*n_{\mathrm{pca}}$ channels. For missing or located on the border spots, we apply padding by taking the median expression over each channel in ${\text{spot}}_{i}$.

#### 2.1.4 Clustering

The default clustering algorithm is *Bayesian Gaussian Mixture* with a diagonal covariance matrix from the *sklearn* package, but we also support *K-Means, Leiden*, and *Louvain*. Once the cluster labels are obtained, an additional preprocessing step can be applied. A K-Neighbors Classifier is fitted using spatial coordinates and the already obtained clusters to refine the cluster assignments in spatial space through majority voting.

### 2.2 Model architecture

AESTETIK utilizes a convolutional deep-learning autoencoder with a standard encoder–decoder architecture and a bottleneck layer. The encoder comprises a convolutional layer, max-pooling layer, batch normalization, *ReLU* activation, and linear layer. Default hyperparameters include 64 convolutional kernels (size 7), dropout (p=0.3), max-pooling (stride 3), and a linear layer (size 16). The decoder follows a mirrored architecture, concluding with a sigmoid function to constrain output values in the range [0,1]. AESTETIK is a *Python* package implemented in *PyTorch*.

#### 2.2.1 Autoencoder ensemble

To improve the network stability, we use ensemble architectures for both the encoder and decoder, utilizing random *LeCun* (LeCun *et al.* 1989) initialization. The ensemble’s output is determined by taking the median over predictions. We train an ensemble with 3 encoders and decoders. The final representation is computed by dropout sampling 1000 times and taking the median value.

#### 2.2.2 Reconstruction loss

We use a reconstruction loss to ensure that the latent space effectively captures the biological complexity of the morphology and transcriptomics modalities. We define it as:


(2) $$L_{\mathrm{rec}}=\alpha *L_{rec_{m}}+(3-\alpha )*L_{rec_{tr}},$$

where α∈[0,3] is a hyperparameter for the morphology weight. $L_{rec_{\left[m,tr\right]}}$ is the standard L1 reconstruction loss with *m* for morphology and *tr* for transcriptomics modality. We use L1 loss due to the input-output range being [0,1].

#### 2.2.3 Multi-triplet loss

We apply triplet loss to preserve the structure across modalities. Its primary objective is to learn a spot representation in which similar instances are closer together, while dissimilar instances are farther apart. Define an anchor point *A* with label $l_{i}$, then we draw at random a positive point *P* with label $l_{i}$ and a negative point *N* with label $l_{j}$ such that $l_{i}\neq l_{j}$, then the single triplet loss is defined as:


$$L_{\mathrm{triplet}}(A,P,N)=\max(0,∥f(A)-f(P){∥}^{2}-∥f(A)-f(N){∥}^{2}+\mathrm{margin})$$

In spatial transcriptomics, multiple classes and high noise ratios are typical. Using just one positive and negative point can lead to unstable representation and increase the training time due to alternations. To improve the spot representation robustness, we propose the multi-triplet loss motivated by Sohn (2016). Let *L* be the number of unique labels in the dataset. Define an anchor point *A* with label $l_{i}$, then we draw, with replacement, L−1 positive points $\left\{P_{1},P_{2},\ldots ,P_{L-1}\right\}$ with label $l_{i}$. Additionally, for each label $l_{j}$ where j≠i, we draw a single negative point, resulting in $\left\{N_{1},N_{2},\ldots ,N_{L-1}\right\}$. Then the multi-triplet loss for a single modality can be defined as:


$$L_{\mathrm{multi}\_\mathrm{triplet}}(A)=\frac{1}{L-1}\sum_{k=1}^{L-1}L_{\mathrm{triplet}}(A,P_{k},N_{k})$$

which when extended to all spots:


$$L_{\mathrm{multi}\_\mathrm{triplet}}=\frac{1}{N_{\mathrm{spots}}}\sum_{l=1}^{N_{\mathrm{spots}}}L_{\mathrm{multi}\_\mathrm{triplet}}(A_{l})$$

The modality-weighted multi-triplet loss is defined as:


(3) $$L_{\mathrm{multi}\_\mathrm{triplet}}=\alpha *L_{\mathrm{multi}\_\mathrm{triple}t_{m}}+(3-\alpha )*L_{\mathrm{multi}\_\mathrm{triple}t_{tr}}$$

with α defined as in Equation (2).

#### 2.2.4 Loss function

The overall loss function for training combines reconstruction loss to ensure accurate latent representation and multi-triplet loss for preserving structure across modalities. Formally, it is defined as:


$$L_{\mathrm{AESTETIK}}=L_{\mathrm{rec}}+L_{\mathrm{multi}\_\mathrm{triplet}},$$

which can be rewritten as:


(4) $$\begin{array}{cc} L_{\mathrm{AESTETIK}}=\alpha *(L_{rec_{m}}+L_{\mathrm{multi}\_\mathrm{triple}t_{m}}) & \\ +(3-\alpha )*(L_{rec_{tr}}+L_{\mathrm{multi}\_\mathrm{triple}t_{tr}}) & \end{array}$$

where the user-defined α∈[0,3] controls the mixture between modalities: α=0 uses only the transcriptomics modality, α=3 uses only the morphology modality, and intermediate values yield mixed contributions. This weighting can also be rescaled to custom ranges if desired.

#### 2.2.5 Training details

The model is trained for 100 epochs using *Adam* (Kingma and Ba 2014) with a weight decay of 1e−6, a learning rate of 1e−3, and a batch size corresponding to the number of spots in a tissue slice. The run time is approximately 8 min on a GPU, with inference time under a minute. Computational data analysis was performed at Leonhard Med (https://sis.id.ethz.ch/services/sensitiveresearchdata/) secure trusted research environment at ETH Zurich.

#### 2.2.6 Evaluation

We propose reversed leave-one-out cross-validation for model evaluation to avoid hyperparameter tuning on test samples. Specifically, for each model [including our proposed method and all baseline methods such as GraphST, STAGATE, stLearn, MUSE, PearlST (Wang *et al.* 2024), etc.], we utilize a single sample and its replicates to select hyperparameters through a grid search, aiming to maximize the median ARI. Subsequently, the optimal hyperparameters are applied to the remaining test samples. For state-of-the-art methods, we consider hyperparameter values discussed in the corresponding papers and public code repositories. Hyperparameter values are provided in Table S1, available as supplementary data at *Bioinformatics* online.

To ensure comparable conditions across models, the number of clusters is pre-defined based on the provided ground truth. Performance is assessed using the ARI between ground-truth labels and cluster assignments. We bootstrap 1000 times from the median ARI across the test folds and report the resulting median ARI and its standard error. For most datasets, we generated all possible sample combinations (folds). However, for the larger CosMx NanoString™ Liver dataset we use each FOV to select the hyperparameters, but we evaluate on randomly selected 20 FOVs, not adjacent to the FOVs used for optimization.

#### 2.2.7 Ablation study

In the ablation study on AESTETIK, we adhere to the procedure described in Section 2.2.6, where we fix the value for the hyperparameter of interest and assess its impact on the ARI. To ensure comparability across datasets, we compute an ARI z-score.

### 2.3 Downstream applications

#### 2.3.1 Marker genes

For marker genes, we use the *rank genes groups* function from *scanpy* using the Wilcoxon signed-rank test. Significant marker genes (adjusted *P*-value < .05) are selected and sorted by their average log-fold change. The top 15 marker genes per cluster are reported.

#### 2.3.2 Pathway analysis

For pathway analysis, we utilize the multivariate linear model from the *decoupler* package (Badia-i-Mompel *et al.* 2022) to compute regulatory pathway activities from the *PROGENy* database (Schubert *et al.* 2018).

#### 2.3.3 Cluster centroids in latent space

To determine the centroid for each cluster in the latent space, we used a method minimizing the sum of Euclidean distances among all samples within that class. Subsequently, we computed the top N spots near each cluster centroid.

## 3 Results

### 3.1 AESTETIK integrates spatial, transcriptomics, and morphology information

We introduce **A**uto**E**ncoder for **S**patial **T**ranscriptomics **E**xpression with **T**opology and **I**mage **K**nowledge (AESTETIK), a convolutional autoencoder model (Fig. 1B). It jointly integrates transcriptomics and morphology information at a spot level and topology at a neighborhood level to learn accurate spot representations that capture biological complexity.

Firstly, we preprocess the transcriptomics profiles and apply principal component analysis (PCA) (Lun *et al.* 2016). Simultaneously, the pre-trained on *Imagenet* (Deng *et al.* 2009) deep-learning model, *Inception v3* (Szegedy *et al.* 2016), is used to extract morphology spot features, followed by PCA. After computing clusters separately to preserve the modality-specific structure, we concatenate the top principal components (PC) from both modalities. Next, we construct a square grid for each spot that includes spatially neighboring spots. This grid per spot, along with the precomputed clusters, serves as an input to AESTETIK. The model relies on a convolutional encoder–decoder architecture (Fig. 1C) to learn accurate spatial-, transcriptomics-, and morphology-informed spot representations. Ultimately, the learned representations can be leveraged for various downstream applications, including but not limited to clustering, gene expression, morphology, and pathway analysis.

The motivation for the grid construction is to form an image-like representation, with grid encoding for spatial neighborhood and channels for both transcriptomics and morphology modalities. We frame the machine-learning problem as image pattern recognition and compression, with convolutional autoencoders being the state-of-the-art architecture for addressing these challenges (Pramerdorfer and Kampel 2016, Voulodimos *et al.* 2018). The bottleneck layer serves as a constriction for information flow, forcing the model to capture the biological signal. Moreover, AESTETIK’s loss function [Equation (4)] is designed to optimize multiple objectives simultaneously by combining reconstruction loss for accurate latent representation and multi-triplet loss [Fig. 1D, Equation (3)] for structure preservation across modalities. This dual optimization ensures a comprehensive and informative representation of each data modality.

### 3.2 AESTETIK improves the identification of spatial domains

**Figure 2:**
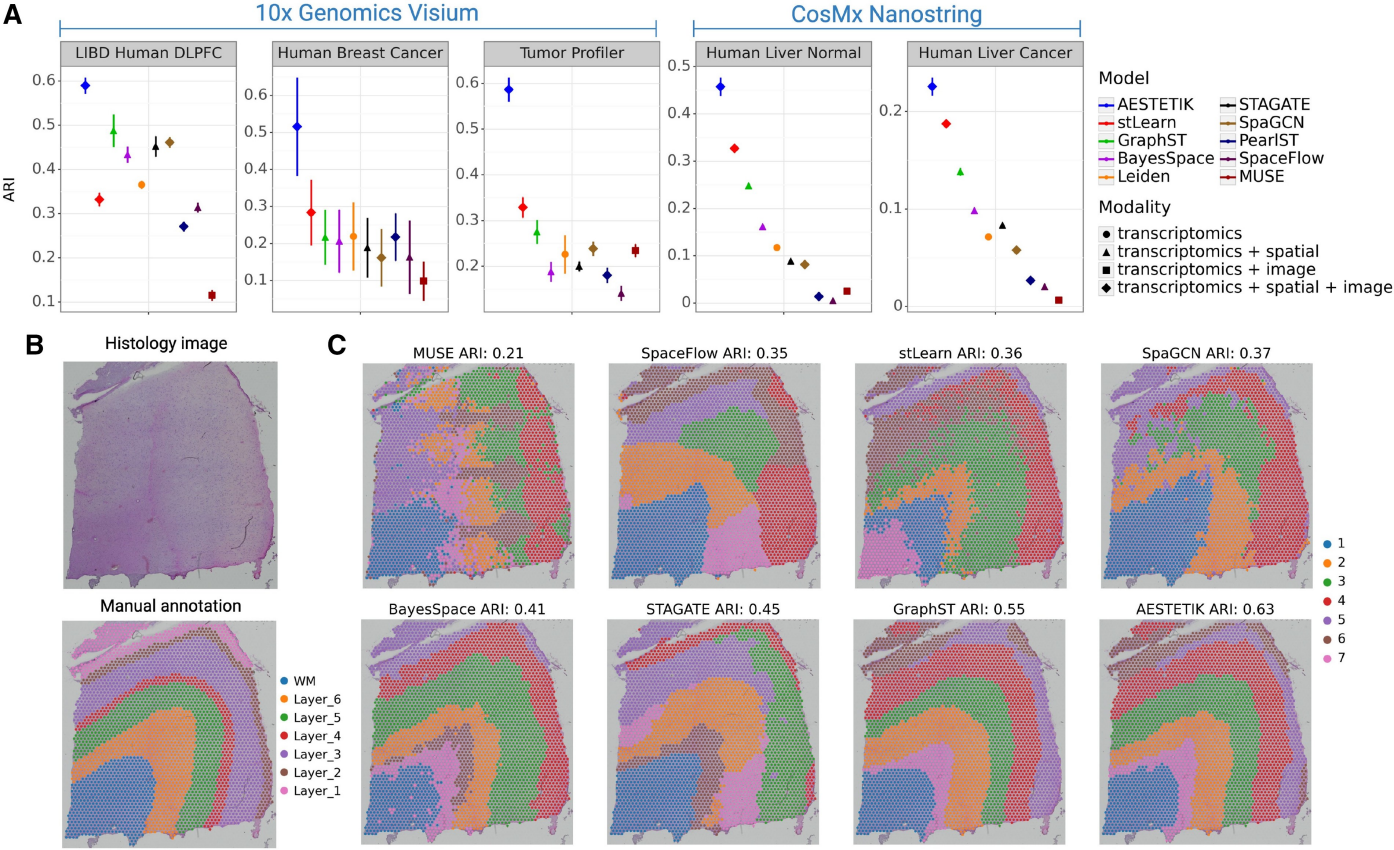
AESTETIK improves the identification of spatial domains with coherent expression and morphology. (A) Benchmark of AESTETIK and previous state-of-the-art methods in spatial transcriptomics on five datasets across two technology platforms. The *y*-axis represents the ARI between the ground truth and the predicted labels. Models are ordered based on their relative rank across the datasets. The shape represents the modalities the model integrates. (B) Histology image and manual annotation of slice 151676 from the LIBD human DLPFC dataset (Maynard *et al*. 2021) and (C) Comparison of cluster assignments for the same slice.

We benchmarked AESTETIK performance on multiple datasets with available ground truth annotations (Fig. 2A). In line with the methodology of Zhao *et al.* (2021), Bao *et al.* (2022), Pham *et al.* (2023), Hu *et al.* (2021), Dong and Zhang (2022), Long *et al.* (2023), we adopted the Adjusted Rand Index (ARI) to measure the similarity between predicted cluster labels and ground truth, with the number of clusters set to match that in the ground truth. To avoid hyperparameter tuning on the samples used for testing, we introduced reversed leave-one-out cross-validation. More specifically, we used a single sample and its replicates to select hyperparameters to maximize the median ARI. Then, the optimal hyperparameters were applied to the remaining test samples. This process was iterated over all folds, and the resulting median ARI, along with the standard error, is reported (Fig. 2A). Additional clustering metrics, including adjusted mutual information, completeness score, and the Fowlkes–Mallows index, are presented in Fig. S1, available as supplementary data at *Bioinformatics* online and yield results consistent with the ARI, supporting the robustness of the clustering performance.

AESTETIK consistently yielded substantially improved cluster assignments closer to the ground truth annotations over previous state-of-the-art methods across all datasets (Fig. 2A). For example, on the LIBD Human DLPFC dataset (Maynard *et al.* 2021), AESTETIK achieved the highest ARI of 0.59±0.02, significantly surpassing the second best model—*GraphST—*by 21%. The LIBD Human DLPFC dataset comprises 12 tissue slices obtained from the dorsolateral prefrontal cortex (DLPFC) brain region, sequenced using Visium from 10x Genomics™, together with curated manual annotations based on brain cytoarchitecture and known marker genes (Fig. 2B). This improvement highlights the superior performance of AESTETIK in effectively integrating the spatial modality and generating accurate cluster assignments in structured brain tissue. *SpaGCN* and *GraphST* demonstrated lower performance, achieving ARI of 0.46±0.02 and 0.48±0.03, respectively. We note that some methods, such as *STAGATE* and *DeepST* (Dong and Zhang 2022, Xu *et al.* 2022a), report ARI values around 0.6 on individual DLPFC slices. However, these results are typically obtained by optimizing and evaluating on the same slice, whereas our cross-validated protocol (Section 2.2.6) trains on held-out patients and evaluates on unseen samples, measuring generalization rather than within-slice fitting.

To qualitatively illustrate the cluster assignments, we compare them for slice 151676 (Fig. 2C), using the closest annotations to the ground truth across folds. *MUSE* (ARI 0.23), *SpaceFlow* (ARI 0.35), *stLearn* (ARI 0.36), and *SpaGCN* (ARI 0.37) mixed the brain layers, accompanied by noise along the boundaries. *BayesSpace* (ARI 0.40) partitioned the white matter (WM) and layer 6 into multiple groups. While *GraphST* (ARI 0.55) generated mostly well-defined clusters, layers 1, 2, and 3 were inconsistent. Notably, AESTETIK (ARI 0.63) identified the brain architecture, and its clusters displayed clearer definitions at the boundaries, leading to superior performance (Fig. 2; Fig. S2, available as supplementary data at *Bioinformatics* online).

Next, we investigate the methods’ performance on the Human Breast Cancer dataset, which comprises 5 tissue slices sequenced using Visium from 10x Genomics™ and annotated independently in two different labs (Wu *et al.* 2021). This dataset presents unique challenges, primarily stemming from the considerable inter- and intra-sample heterogeneity, including variations in the cancer cell population. AESTETIK achieved the closest clusters to the ground truth labels with an ARI of 0.52±0.13 (Fig. 2A), indicating a 2-fold increase over the second best model, *stLearn* (ARI 0.27±0.09). Despite wide standard error intervals observed in all models, AESTETIK exhibited heightened robustness, surpassing the challenges posed by the complexity of breast cancer tissue (Fig. S3, available as supplementary data at *Bioinformatics* online).

**Figure 3:**
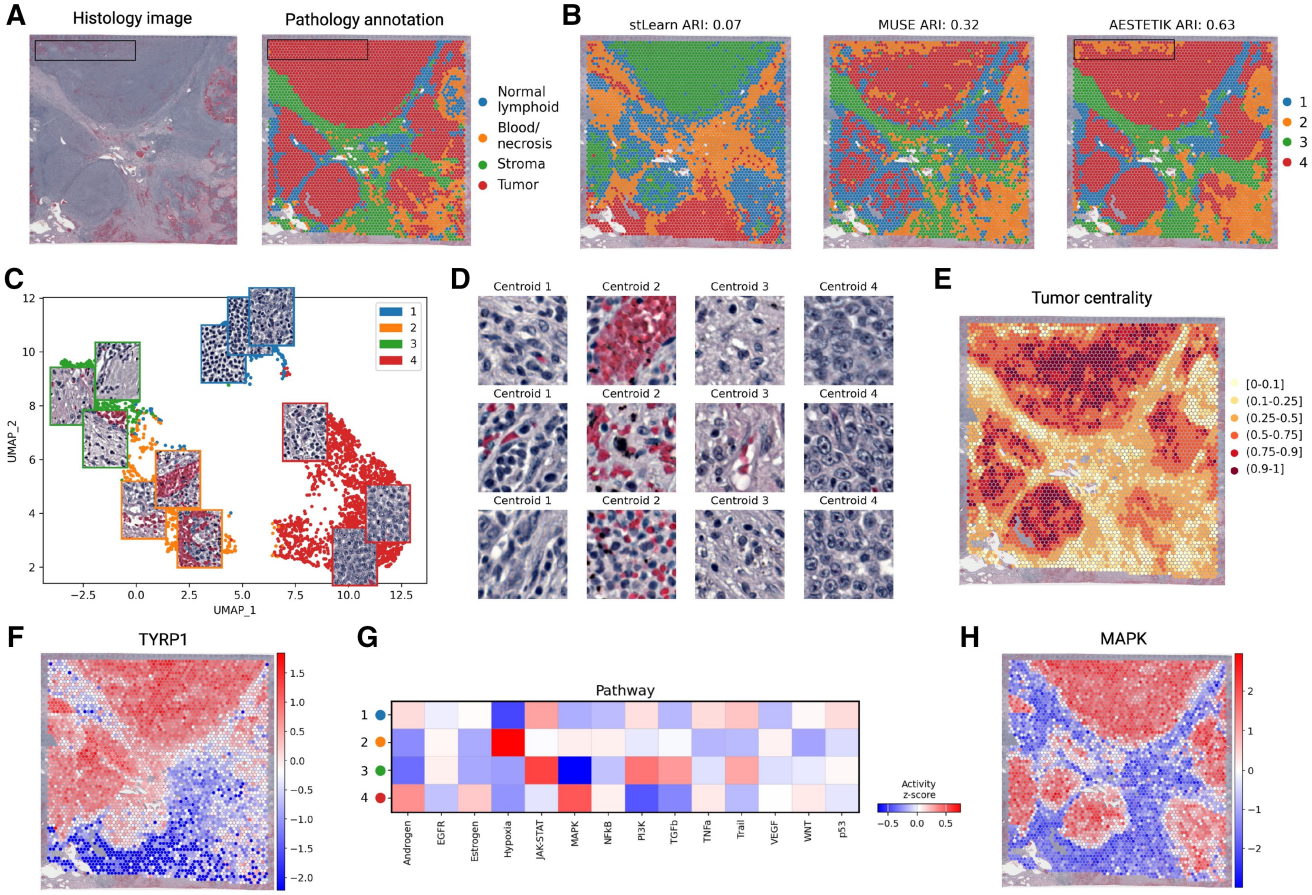
AESTETIK effectively incorporates the morphology modality, revealing biologically relevant spatial organization of cancer tissue. (A) Histology image and pathology annotation of slice MACEGEJ-2–2 from the Tumor Profiler dataset. (B) Comparison of cluster assignments for slice MACEGEJ-2–2. (C) UMAP plot of AESTETIK’s latent space with randomly sampled spot images. (D) Most representative cluster spots based on the obtained representations. (E) Euclidean distance in latent space of each spot to the tumor centroid plotted in spatial space. Most representative spots are located in the middle of the tumor formations. (F) Spatial marker gene expression of TYRP1. (G) Pathway analysis of the identified clusters using *decoupler* (Badia-i-Mompel *et al*. 2022). (H) Spatial activation of MAPK pathway.

### 3.3 AESTETIK effectively incorporates the morphology modality

We introduce a new and yet unreleased spatial transcriptomics dataset with 9 distinct tissue regions sequenced using Visium from 10x Genomics™ from the Tumor Profiler study (Irmisch *et al.* 2021). Each region has a replicate resulting in 18 samples of size $6.5\times 6.5\;{\text{mm}}^{2}$, with data including 10x Genomics™ *Space Ranger v3.0.0* outputs and a corresponding H&E image scanned at a high resolution of 0.3 μm/pixel. The tissue regions originate from seven patients with metastatic melanoma, each characterized by one of the following immune subtypes: immune desert, immune excluded, or inflamed. The ground truth annotations were obtained using histopathology software [HALO AI™ (Indica Labs, Corrales, NM, USA)], classifying the spots into one of the following categories: tumor, stroma, normal lymphoid, and blood/necrosis. Following this, a pathologist manually reviewed the model predictions (Fig. 3A). We consider this dataset to be a valuable reference benchmark for evaluating the performance of spatial transcriptomics models, particularly in terms of their ability to integrate morphology effectively.

On this dataset, AESTETIK achieved a 79% increase in ARI (0.59±0.03) over previous state-of-the-art methods, demonstrating effective use of the morphology modality (Fig. 2A). While both *stLearn* (ARI 0.33±0.02) and *MUSE* (ARI 0.23±0.02) use the same pre-trained *Inception v3* (Szegedy *et al.* 2016) for extracting morphology features, they fall short in effectively leveraging this information (Fig. 3B). On the other hand, AESTETIK not only produced accurate cluster assignments (Fig. 3B), but also identified a hemorrhage region in the upper left of the H&E image, which was overlooked during annotation (black box in Fig. 3A and B, and Fig. S4, available as supplementary data at *Bioinformatics* online).

**Figure 4:**
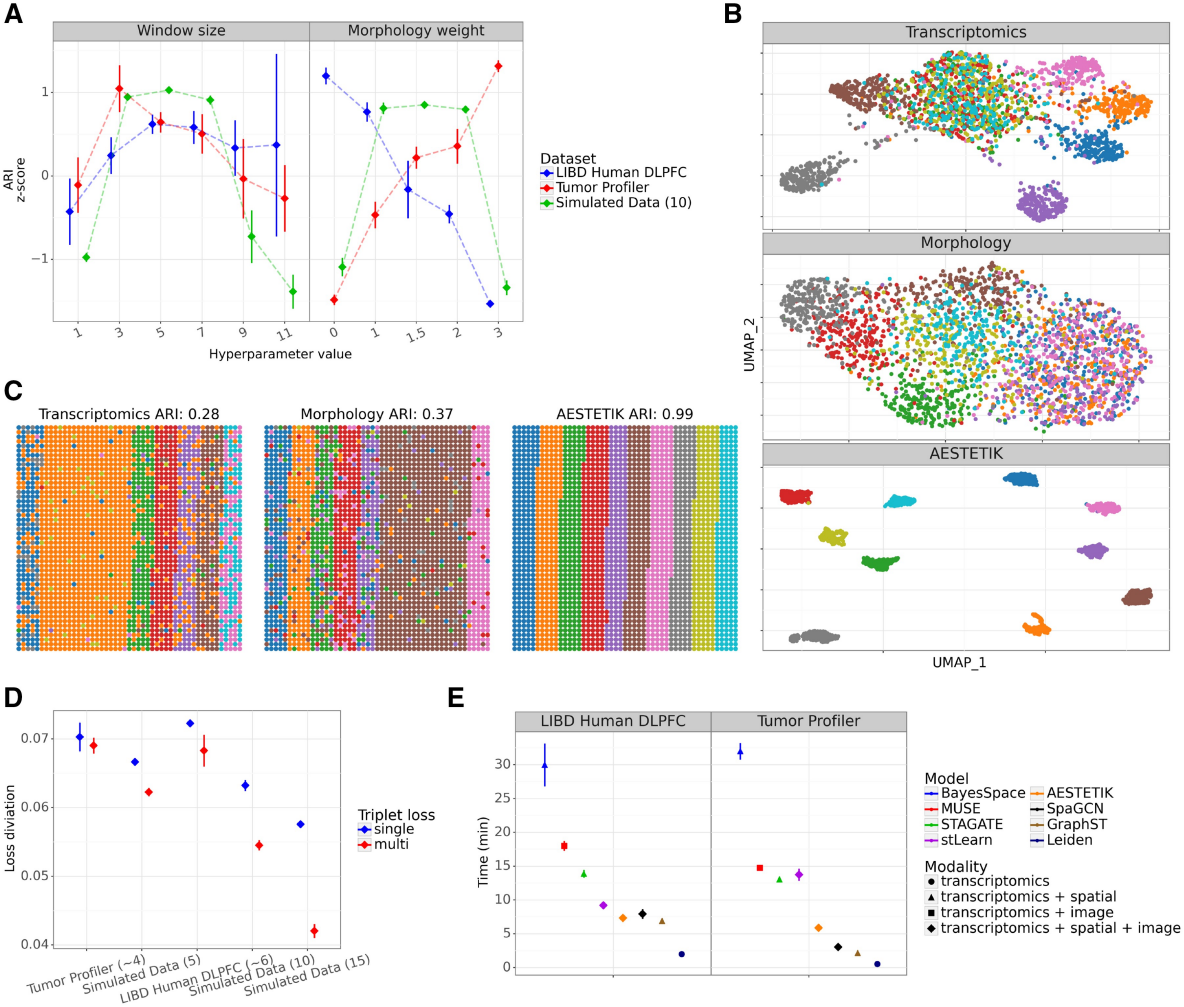
Joint integration of multi-modal data enhances computational analysis. (A) Ablation study on the influence of window size and morphology weight on the ARI. The *y*-axis represents the ARI, normalized by dataset. (B) UMAP visualization of single (transcriptomics, morphology) and combined (AESTETIK) modality representations on a simulated tissue slice, colored by the 10 ground truth annotations. (C) Cluster assignments based on only a single modality (transcriptomics, morphology) and AESTETIK’s joint representations. (D) Comparing the stability of the single and multi-triplet loss on the loss function of AESTETIK across datasets. The number, following the dataset name, is the median number of clusters present. The *y*-axis represents the standard deviation computed on the loss difference over successive training epochs. (E) Runtime for the evaluated clustering methods. The *y*-axis represents the time in minutes. Models are ordered based on their relative rank across the datasets.

Next, we qualitatively explore the latent representations and the identified spatial domains to showcase the utility of the learned multi-modal representations (Fig. 3A). We visualized the latent space using *UMAP* (McInnes *et al.* 2018) with randomly sampled morphology spot representations (Fig. 3C). We observe an aggregation of tumor spots (cluster 4) on the bottom-right side, showcasing similarities in their characteristics. On the lower left side, there are areas with blood and necrosis. While clusters 1 and 3, representing normal lymphoid tissue and stromal cells, are positioned in the upper part, a closer inspection reveals discernible differences in their underlying structures. To further enhance the interpretability of the spot representations, we selected the most representative spots from each cluster (Fig. 3D). Visually, tumor cells within cluster 4 exhibit distinct characteristics; they appear significantly larger, displaying irregular shapes, and possessing enlarged nuclei. Stromal cells (cluster 3) have an elongated morphology and are noticeably more scattered (Manetti 2021) compared to the normal lymphoid cells (cluster 1), which are generally smaller and denser (Van Der Meer *et al.* 2007).

Furthermore, to illustrate the effect of encoding spatial information in latent space, we computed the Euclidean distance of each spot to the tumor centroid and visualized it spatially (Fig. 3E). While stromal cells are the furthest, we observed that tumor cells close in latent space are clustered spatially, with the most representative spots located in the middle of the tumor formations.

To further validate our findings within the context of the learned multi-modal representations, we found TYRP1 and TKTL1 among the top tumor marker genes in the tumor cluster, confirming the model predictions for the spatially resolved identification of melanoma cells (Fig. 3F and Fig. S5, available as supplementary data at *Bioinformatics* online). Notably, the TYRP1 gene is involved in melanocyte pigmentation, associated with melanoma progression and is a target for oncological immunotherapy (Ghanem and Fabrice 2011, Journe *et al.* 2011, Qiu *et al.* 2016). The second highly upregulated gene, TKTL1, is implicated in the progression of melanoma and contributes to the increased invasion of melanoma cells (Jayachandran *et al.* 2016). Further, we performed a pathway analysis of the tumor clusters using *decoupler* (Badia-i-Mompel *et al.* 2022) (Fig. 3G) which revealed increased activity of the MAPK pathway in the cancer cluster (Fig. 3H), known for promoting cell proliferation, invasion, metastasis, migration, survival, and angiogenesis (Inamdar *et al.* 2010, Shain *et al.* 2015, Amaral *et al.* 2017). Furthermore, we observed that hypoxia signatures were predominant in areas of necrosis and hemorrhage and JAK-STAT inflammatory signaling was predominant in the cancer microenvironment clusters 1 and 3. These pathway-level analyses underline the robust associations of the spatially resolved clustering results achieved by AESTETIK and support interpretation in the context of the underlying biology.

### 3.4 AESTETIK improves cluster assignment in single-cell spatial transcriptomics

CosMx NanoString™ released a liver dataset with single-cell resolution, encompassing two tissue regions from normal and cancer patients and capturing 1000 genes. It offers valuable insights into liver biology and cancer characteristics. More specifically, using these datasets, we assess the models’ effectiveness at single-cell resolution by comparing the clusters they produce with the cell types reported by NanoString™. In both normal and cancer liver tissue, AESTETIK exhibited outstanding performance, substantially outperforming the other models by 39% and 21%, with ARI of 0.46±0.02 and 0.23±0.00, respectively (Fig. 2A, Figs S6 and S7, available as supplementary data at *Bioinformatics* online). The second best model, *stLearn*, attained a score of 0.33±0.00 and 0.19±0.00, followed by *GraphST* with 0.24±0.00 and 0.14±0.00. Overall, the clustering accuracy on the cancer tissue is lower compared to the normal sample. However, the relative trend in the ranking of the models remained consistent.

### 3.5 Joint integration of multi-modal data enhances computational analysis

To pinpoint the benefit of the spatial modality in the LIBD Human DLPFC and Tumor Profiler datasets, we systematically varied the grid’s window size, ranging from 1 (w/o spatial information) to 11, and measured the change in ARI (Fig. 4A). The grid size determines the number of spatially adjacent spots to consider. Local spatial information proved important, preserving local details and spot-to-spot variability. However, incorporating a more extensive global context through a larger window size (e.g. 11) introduced noise and hampered performance, which was likely due to signal over-smoothing and the loss of spot-specific details.

Similarly, we studied the contribution of each modality by varying the morphology weight (0—no morphology; 1.5—equal weight between transcriptomics and morphology; 3—only morphology). As expected, we found that the transcriptomics modality in the brain dataset is informative given the provided ground truth annotations, relying on known cytoarchitecture and marker genes (Maynard *et al.* 2021). In contrast, the ground truth for the Tumor Profiler, derived from histopathology software, was morphology driven (Fig. 4A). Importantly, our method can be applied to datasets lacking histological images. By setting the morphology weight to 0, the method relies exclusively on transcriptomics data, highlighting the framework’s flexibility and its compatibility with platforms such as Stereo-seq, Slide-seq V2, and MERFISH.

Furthermore, to underscore the significance of methods incorporating all data modalities, we present a scenario illustrating the necessity of both modalities to reveal ground truth annotations (Fig. 4B). Following the approach of Bao *et al.* (2022), we simulated data where both modalities are essential for accurate cluster identification. Our ablation study demonstrated that the optimal ARI was achieved when accounting for both modalities, thus emphasizing the critical significance of multi-modal data integration (Fig. 4C).

Additionally, the multi-triplet loss demonstrated an enhancement in loss stability during training compared to the single triplet loss (Fig. 4D). The refined positioning of clusters in latent space, considering multiple positive and negative spots, becomes crucial, especially when dealing with datasets containing numerous clusters. Lastly, the runtime per tissue slice for our model, incorporating all three modalities (∼8 min), was either lower or comparable to that of other models (Fig. 4E). For example, *BayesSpace*, *MUSE*, and *STAGATE*, incorporating only two modalities, required ∼28, ∼17, and ∼13 min, respectively. Moreover, we demonstrate that AESTETIK is well-suited for analyzing large spatial transcriptomics datasets, scaling to millions of spots (Fig. S9, available as supplementary data at *Bioinformatics* online).

## 4 Discussion

In this work, we propose AESTETIK, a method that jointly integrates spatial, transcriptomics, and morphology information to learn accurate spot representations. Our results consistently showed superior performance to state-of-the-art methods across structured tissues (e.g. brain) and cancer tissues with heterogeneous cell populations (e.g. breast, melanoma, liver) across widely adopted spatial transcriptomics technologies at both spot-level (e.g. 10x Genomics™, Visium) and single-cell-level resolution (e.g. CosMx NanoString™). Furthermore, by setting the morphology weight α=0, we validated that our framework generalizes to imaging-based spatial transcriptomics platforms without histological images, such as MERFISH (Moffitt *et al.* 2018), where the model operates using transcriptomics and spatial information alone and yields accurate spatial domain assignments (Fig. S8, available as supplementary data at *Bioinformatics* online). Additionally, to emphasize the importance of spatial multimodal integration, we systematically showed that joint integration of multiple data modalities significantly enhances spatial transcriptomics analysis and leads to more accurate spot annotations.

This improvement in spot representation resulted from modeling the spatial transcriptomics modalities as a grid encoding the spatial spot neighborhood and channels as transcriptomics and morphology modalities. Our approach framed the machine-learning problem as image pattern recognition and compression, where convolution filters jointly learn the importance of neighboring spots and channels. This proved beneficial in both structured and heterogeneous tissues.

In contrast, GNN-based methods such as *SpaGCN*, *GraphST*, and *STAGATE* show variable performance across tissue types and spatial transcriptomics technologies. This variability can be attributed to their susceptibility to noise (Dai *et al.* 2018, Zügner *et al.* 2018). Graph structures propagate information through edges connecting neighboring spots, which is advantageous in structured tissues (e.g. brain) with coherent spatial patterns. However, in cells with lower sequencing quality (e.g. sequencing dropouts, measurement errors, or heterogeneous populations) or in highly heterogeneous tissues (e.g. cancer), noisy nodes or mis-specified edges can propagate errors and amplify the influence of outliers, reducing robustness. Standard graph convolutional methods (e.g. *SpaGCN*, *GraphST*) rely on a fixed graph topology derived from spatial proximity, meaning that once edges are constructed, the aggregation scheme cannot adapt to local tissue heterogeneity; any mis-specified edge will persistently propagate noise across training. Attention-based graph methods (e.g. *STAGATE*) attempt to address this by learning feature-dependent edge weights, but introduce different vulnerabilities: attention weight collapse toward noisy or outlier nodes when the feature signal-to-noise ratio is low (Dai *et al.* 2018), and overfitting to spurious pairwise feature correlations in high-noise regimes, leading to unstable embeddings. By comparison, our convolutional approach imposes a regular, translation-invariant grid neighborhood, where shared convolutional kernels jointly learn the importance of neighboring spots and modality channels. This design has two key advantages. First, the aggregation topology is deterministic and decoupled from the feature space: unlike graph attention, the neighborhood structure does not depend on spot features, avoiding the failure modes described above. Second, because the same kernels are applied across all spatial positions, the model generalizes local patterns rather than fitting spot-specific noise. For independent noise, uniform aggregation over a fixed *k*-neighborhood reduces variance by a factor of 1/k; learned convolutional kernels can further improve on this by down-weighting uninformative channels, while still maintaining the stability that comes from a fixed, shared aggregation structure.

More broadly, several recent approaches have been developed for spatial domain identification and multimodal integration. Among graph-based methods, *DeepST* (Xu *et al.* 2022a) combines a graph neural network with a denoising autoencoder, while *SpaceFlow* (Ren *et al.* 2022) uses a deep graph network with spatially regularized embeddings to model tissue dynamics. Beyond graph-based approaches, *SpaMask* (Min *et al.* 2025) leverages masked autoencoding to capture local spatial context, and *SpaCross* (Fang and Min 2025) uses a cross-masked latent consistency module to reinforce implicit constraints on the latent space. Other recent methods, including *SpaBatch* (Niu *et al.* 2025a), *SpaICL* (Zhao and Min 2025), *SpatialCVGAE* (Niu *et al.* 2025b), and *STG3Net* (Fang *et al.* 2024), address related challenges such as batch correction across spatial slices, contrastive self-supervised learning, variational graph inference, and graph-transformer architectures for spatial domains. While each offers specialized advantages, AESTETIK differs by framing spatial transcriptomics as an image-like grid representation that simultaneously captures local spatial neighborhoods and multiple modalities (transcriptomics + morphology) through convolutional filters, providing an efficient and generalizable approach across both structured and heterogeneous tissues.

Further, in our ablation study on the brain dataset, we quantitatively demonstrated the significance of the spatial modality in identifying the brain layer structure. We discovered that a relatively small grid’s window size (5–7) sufficiently captures the desired spatial signal. Opting for a larger neighborhood (e.g. window size 11) offers no extra value. Unlike the global tissue context, the local environment better preserves spot-specific signals and nearby variability. Ultimately, our ablation results underscore (i) the importance of jointly integrating the available spatial transcriptomics data modalities for accurate spot representation, and (ii) the necessity for external knowledge to prioritize the signal of interest, depending on the particular research question at hand.

Several paths to further improve model accuracy appear promising. (i) We used the pre-trained *Inception v3* (Szegedy *et al.* 2016) to extract morphology features. However, adopting a model tailored to a specific task (e.g. cell nuclei segmentation and classification) would likely yield more informative spot features, potentially leading to improved overall performance. (ii) AESTETIK randomly selects the positive and negative pairs for each anchor point during training. We believe this process can be improved by utilizing a *smarter* strategy for triplet mining, which would eventually improve the performance and robustness to noise.

In the future, AESTETIK could be effectively applied to fine-map cell populations in spatial transcriptomics datasets (Fan *et al.* 2020, Xu *et al.* 2022b, Zheng *et al.* 2023), to systematically analyze the interplay between different modalities by varying their contribution and to gain a multi-modal understanding of key biological processes. More broadly, the learned latent representations are general-purpose embeddings that can serve as input to a range of downstream analyses beyond spatial domain identification, including gene expression imputation, cell–cell communication inference, and trajectory analysis. As demonstrated on the melanoma dataset (Fig. 3), the representations already support marker gene identification, pathway activity scoring, and morphology-based cluster interpretation, illustrating their utility for tumor microenvironment characterization. To foster these downstream applications, we have released the code for AESTETIK along with examples demonstrating its usage. Moreover, we anticipate that upon its release, the 10x Genomics™, Visium dataset from the Tumor Profiler study will serve as a valuable reference benchmark for assessing spatial transcriptomics model performance and explainability. Thus, we hope that our model, together with this dataset, will stimulate further improvements in computational spatial transcriptomics analysis.

## Supplementary Material

btag316_Supplementary_Data

## Data Availability

The LIBD Human DLPFC dataset is available at https://github.com/LieberInstitute/HumanPilot and http://research.libd.org/spatialLIBD; Human Breast Cancer—Zenodo https://doi.org/10.5281/zenodo.4739739; Human Liver Normal and Cancer – https://nanostring.com/products/cosmx-spatial-molecular-imager/human-liver-rna-ffpe-dataset/; MERFISH Mouse Hypothalamus—Dryad https://doi.org/10.5061/dryad.8t8s248 (Moffitt *et al.* 2018). The metastatic melanoma dataset comprises 18 tissue slices from Tumor Profiler Study samples sequenced using the Visium platform (10x Genomics). As these data contain sensitive clinical information and are subject to ethical and privacy restrictions, they cannot be released without restriction. The data are accessible upon request through the Tumor Profiler Consortium's portal at https://tumorprofilercenter.ch/contacts. Requests must include a brief scientific proposal outlining the intended use. The Consortium typically reviews requests within four to six weeks and determines the scope, duration and conditions of data access. Approved users are required to comply with data use agreements that restrict data use to specified research purposes and prohibit further sharing without authorization

## Tumor Profiler Consortium

Rudolf Aebersold^5^, Melike Ak^33^, Faisal S Al-Quaddoomi^12,22^, Silvana I Albert^10^, Jonas Albinus^10^, Ilaria Alborelli^29^, Sonali Andani^9,22,31,36^, Per-Olof Attinger^14^, Marina Bacac^21^, Daniel Baumhoer^29^, Beatrice Beck-Schimmer^44^, Niko Beerenwinkel^7,22^, Christian Beisel^7^, Lara Bernasconi^32^, Anne Bertolini^12,22^, Bernd Bodenmiller^11,40^, Ximena Bonilla^9^, Lars Bosshard^12,22^, Byron Calgua^29^, Ruben Casanova^40^, Stéphane Chevrier^40^, Natalia Chicherova^12,22^, Ricardo Coelho^23^, Maya D’Costa^13^, Esther Danenberg^42^, Natalie R Davidson^9^, Monica-Andreea Dragan^7^, Reinhard Dummer^33^, Stefanie Engler^40^, Martin Erkens^19^, Katja Eschbach^7^, Cinzia Esposito^42^, André Fedier^23^, Pedro F Ferreira^7^, Joanna Ficek-Pascual^1,9,16,22,31^, Anja L Frei^36^, Bruno Frey^18^, Sandra Goetze^10^, Linda Grob^12,22^, Gabriele Gut^42^, Detlef Günther^8^, Pirmin Haeuptle^3^, Viola Heinzelmann-Schwarz^23,28^, Sylvia Herter^21^, Rene Holtackers^42^, Tamara Huesser^21^, Alexander Immer^9,17^, Anja Irmisch^33^, Francis Jacob^23^, Andrea Jacobs^40^, Tim M Jaeger^14^, Katharina Jahn^7^, Alva R James^9,22,31^, Philip M Jermann^29^, André Kahles^9,22,31^, Abdullah Kahraman^22,36^, Viktor H Koelzer^36,41^, Werner Kuebler^30^, Jack Kuipers^7,22^, Christian P Kunze^27^, Christian Kurzeder^26^, Kjong-Van Lehmann^2,4,9,15^, Mitchell Levesque^33^, Ulrike Lischetti^23^, Flavio C Lombardo^23^, Sebastian Lugert^13^, Gerd Maass^18^, Markus G Manz^35^, Philipp Markolin^9^, Martin Mehnert^10^, Julien Mena^5^, Julian M Metzler^34^, Nicola Miglino^35,41^, Emanuela S Milani^10^, Holger Moch^36^, Simone Muenst^29^, Riccardo Murri^43^, Charlotte KY Ng^29,39^, Stefan Nicolet^29^, Marta Nowak^36^, Monica Nunez Lopez^23^, Patrick GA Pedrioli^6^, Lucas Pelkmans^42^, Salvatore Piscuoglio^23,29^, Michael Prummer^12,22^, Laurie Prélot^9,22,31^, Natalie Rimmer^23^, Mathilde Ritter^23^, Christian Rommel^19^, María L Rosano-González^12,22^, Gunnar Rätsch^1,6,9,22,31^, Natascha Santacroce^7^, Jacobo Sarabia del Castillo^42^, Ramona Schlenker^20^, Petra C Schwalie^19^, Severin Schwan^14^, Tobias Schär^7^, Gabriela Senti^32^, Wenguang Shao^10^, Franziska Singer^12,22^, Sujana Sivapatham^40^, Berend Snijder^5,22^, Bettina Sobottka^36^, Vipin T Sreedharan^12,22^, Stefan Stark^9,22,31^, Daniel J Stekhoven^12,22^, Tanmay Tanna^7,9^, Alexandre PA Theocharides^35^, Tinu M Thomas^9,22,31^, Markus Tolnay^29^, Vinko Tosevski^21^, Nora C Toussaint^12,22^, Mustafa A Tuncel^7,22^, Marina Tusup^33^, Audrey Van Drogen^10^, Marcus Vetter^25^, Tatjana Vlajnic^29^, Sandra Weber^32^, Walter P Weber^24^, Rebekka Wegmann^5^, Michael Weller^38^, Fabian Wendt^10^, Norbert Wey^36^, Andreas Wicki^35,41^, Mattheus HE Wildschut^5,35^, Bernd Wollscheid^10^, Shuqing Yu^12,22^, Johanna Ziegler^33^, Marc Zimmermann^9^, Martin Zoche^36^, Gregor Zuend^37^.

^1^AI Center at ETH Zurich, Andreasstrasse 5, 8092 Zurich, Switzerland. ^2^Cancer Research Center Cologne-Essen, University Hospital Cologne, Cologne, Germany. ^3^Cantonal Hospital Baselland, Medical University Clinic, Rheinstrasse 26, 4410 Liestal, Switzerland. ^4^Center for Integrated Oncology Aachen (CIO-A), Aachen, Germany. ^5^ETH Zurich, Department of Biology, Institute of Molecular Systems Biology, Otto-Stern-Weg 3, 8093 Zurich, Switzerland. ^6^ETH Zurich, Department of Biology, Wolfgang-Pauli-Strasse 27, 8093 Zurich, Switzerland. ^7^ETH Zurich, Department of Biosystems Science and Engineering, Mattenstrasse 26, 4058 Basel, Switzerland. ^8^ETH Zurich, Department of Chemistry and Applied Biosciences, Vladimir-Prelog-Weg 1–5/10, 8093 Zurich, Switzerland. ^9^ETH Zurich, Department of Computer Science, Institute of Machine Learning, Universitätstrasse 6, 8092 Zurich, Switzerland. ^10^ETH Zurich, Department of Health Sciences and Technology, Otto-Stern-Weg 3, 8093 Zurich, Switzerland. ^11^ETH Zurich, Institute of Molecular Health Sciences, Otto-Stern-Weg 7, 8093 Zurich, Switzerland. ^12^ETH Zurich, NEXUS Personalized Health Technologies, Wagistrasse 18, 8952 Zurich, Switzerland. ^13^F. Hoffmann-La Roche Ltd, Grenzacherstrasse 124, 4070 Basel, Switzerland. ^14^F. Hoffmann-La Roche Ltd, Grenzacherstrasse 124, 4070 Basel, Switzerland. ^15^Joint Research Center Computational Biomedicine, University Hospital RWTH Aachen, Aachen, Germany. ^16^Life Science Zurich Graduate School, Biomedicine PhD Program, Winterthurerstrasse 190, 8057 Zurich, Switzerland. ^17^Max Planck ETH Center for Learning Systems. ^18^Roche Diagnostics GmbH, Nonnenwald 2, 82377 Penzberg, Germany. ^19^Roche Pharmaceutical Research and Early Development, Roche Innovation Center Basel, Grenzacherstrasse 124, 4070 Basel, Switzerland. ^20^Roche Pharmaceutical Research and Early Development, Roche Innovation Center Munich, Roche Diagnostics GmbH, Nonnenwald 2, 82377 Penzberg, Germany. ^21^Roche Pharmaceutical Research and Early Development, Roche Innovation Center Zurich, Wagistrasse 10, 8952 Schlieren, Switzerland. ^22^SIB Swiss Institute of Bioinformatics, Lausanne, Switzerland. ^23^University Hospital Basel and University of Basel, Department of Biomedicine, Hebelstrasse 20, 4031 Basel, Switzerland. ^24^University Hospital Basel and University of Basel, Department of Surgery, Brustzentrum, Spitalstrasse 21, 4031 Basel, Switzerland. ^25^University Hospital Basel, Brustzentrum & Tumorzentrum, Petersgraben 4, 4031 Basel, Switzerland. ^26^University Hospital Basel, Brustzentrum, Spitalstrasse 21, 4031 Basel, Switzerland. ^27^University Hospital Basel, Department of Information- and Communication Technology, Spitalstrasse 26, 4031 Basel, Switzerland. ^28^University Hospital Basel, Gynecological Cancer Center, Spitalstrasse 21, 4031 Basel, Switzerland. ^29^University Hospital Basel, Institute of Medical Genetics and Pathology, Schönbeinstrasse 40, 4031 Basel, Switzerland. ^30^University Hospital Basel, Spitalstrasse 21/Petersgraben 4, 4031 Basel, Switzerland. ^31^University Hospital Zurich, Biomedical Informatics, Schmelzbergstrasse 26, 8006 Zurich, Switzerland. ^32^University Hospital Zurich, Clinical Trials Center, Rämistrasse 100, 8091 Zurich, Switzerland. ^33^University Hospital Zurich, Department of Dermatology, Gloriastrasse 31, 8091 Zurich, Switzerland. ^34^University Hospital Zurich, Department of Gynecology, Frauenklinikstrasse 10, 8091 Zurich, Switzerland. ^35^University Hospital Zurich, Department of Medical Oncology and Hematology, Rämistrasse 100, 8091 Zurich, Switzerland. ^36^University Hospital Zurich, Department of Pathology and Molecular Pathology, Schmelzbergstrasse 12, 8091 Zurich, Switzerland. ^37^University Hospital Zurich, Rämistrasse 100, 8091 Zurich, Switzerland. ^38^University Hospital and University of Zurich, Department of Neurology, Frauenklinikstrasse 26, 8091 Zurich, Switzerland. ^39^University of Bern, Department of BioMedical Research, Murtenstrasse 35, 3008 Bern, Switzerland. ^40^University of Zurich, Department of Quantitative Biomedicine, Winterthurerstrasse 190, 8057 Zurich, Switzerland. ^41^University of Zurich, Faculty of Medicine, Zurich, Switzerland. ^42^University of Zurich, Institute of Molecular Life Sciences, Winterthurerstrasse 190, 8057 Zurich, Switzerland. ^43^University of Zurich, Services and Support for Science IT, Winterthurerstrasse 190, 8057 Zurich, Switzerland. ^44^University of Zurich, VP Medicine, Künstlergasse 15, 8001 Zurich, Switzerland.

## Code availability

The open-source implementation of AESTETIK along with a tutorial is available at www.github.com/ratschlab/aestetik as well as on Zenodo with DOI https://zenodo.org/records/18326858. The Snakemake pipeline for reproducing the results is available at www.github.com/ratschlab/st-rep.
