# Supplementary material for: Representation learning for multi-modal spatially resolved transcriptomics data
Source: Bioinformatics. 2026 May 21;42(7):btag316. doi: 10.1093/bioinformatics/btag316 (PMC13371760; doi:10.1093/bioinformatics/btag316)
Supplement: btag316_Supplementary_Data [file btag316_supplementary_data.pdf]

Bioinformatics, 2026, pp. 1–9
10.1101/2024.06.04.24308256
Advance Access Publication Date: 12 May 2026
Supplementary Information

Supplementary Information

Representation learning for multi-modal spatially resolved transcriptomics data

Kalin Nonchev,[1,2,*] Sonali Andani,[1,2,3,†] Joanna Ficek-Pascual,[1,2,†] Marta Nowak,[3] Bettina Sobottka,[3] Tumor Profiler Consortium, Viktor H. Koelzer[3,4,5,*,‡] and Gunnar Rätsch[1,2,6,7,*,‡]

[1]Department of Computer Science, ETH Zurich, Zurich, Switzerland
[2]Swiss Institute of Bioinformatics, Zurich, Switzerland
[3]Computational and Translational Pathology Group, Department of Pathology and Molecular Pathology, University Hospital Zurich, University of Zürich, Zurich, Switzerland
[4]Department of Oncology, University of Oxford, Oxford, United Kingdom
[5]Institute of Medical Genetics and Pathology, University Hospital Basel, Basel, Switzerland
[6]AI Center, ETH Zurich, Zurich, Switzerland
[7]Medical Informatics Unit, University Hospital Zurich, Zurich, Switzerland

*Corresponding authors. kalin.nonchev@inf.ethz.ch; viktor.koelzer@usb.ch; gunnar.raetsch@inf.ethz.ch.
† Sonali Andani and Joanna Ficek-Pascual contributed equally to this work.
‡ Viktor H. Koelzer and Gunnar Rätsch jointly supervised this work.

Abstract

This document contains supplementary methods, tables, and figures for the manuscript *Representation learning for multi-modal spatially resolved transcriptomics data*. References cited here correspond to the bibliography of the main article.

Keywords supplementary methods, supplementary tables, supplementary figures

S1. Comparison with other methods

For comparing other spatial transcriptomics methods, we applied the suggested preprocessing pipelines outlined in the corresponding papers and tutorials. A summary can be found here:

- *BayesSpace* (Zhao et al. 2021): Raw counts are normalized, log1p transformed, the 2000 most variable genes are selected and PCA is applied to select the first 15 PCs.
- *stLearn* (Pham et al. 2023): Raw counts are normalized, log1p transformed, and PCA is applied to select the first 15 PCs. For morphology features *Inception v3* is used.
- *GraphST* (Long et al. 2023): The first 3000 most variable genes are selected in *Seurat v3* style. Then the raw counts are normalized, log1p transformed and scaled.
- *STAGATE* (Dong and Zhang 2022): The first 3000 most variable genes are selected in *Seurat v3* style. Then the raw counts are normalized and log1p transformed.
- *MUSE* (Bao et al. 2022): The first 500 most variable genes are selected in *Seurat v3* style. Then the raw counts are normalized, log1p transformed and PCA is applied to select the first 15 PCs. For morphology features *Inception v3* is used.
- *SpaGCN* (Hu et al. 2021): The MT and ERCC genes are removed along with genes expressed in less than 3 cells. Then the raw counts are normalized, log1p transformed and PCA is applied to select the first 50 PCs.
- *Leiden*: The genes with normalized variance computed in *Seurat v3* style larger than 1 are selected, normalized, log1p transformed, scaled and PCA is applied to select the first 15 PCs.
- *SpaceFlow* (Ren et al. 2022): The genes with normalized variance computed in *Seurat v3* style larger than 1 are selected, normalized, log1p transformed, scaled and PCA is applied to select the first 15 PCs.
- *PearlST* (Wang et al. 2024) (`https://github.com/SunXQlab/PearlST`): Raw counts are normalized, log1p transformed, and the 2000 most highly variable genes are selected. For morphology features, *Inception v3* is used to ensure a consistent image feature extractor across all methods that incorporate histological information. Gene expression is denoised via PDE-based anisotropic diffusion and augmented using weighted spatial neighbors. A Wasserstein adversarial regularized graph autoencoder (WARGA) is trained on the augmented features to learn latent embeddings, followed by K-Means clustering.

Received: 12 May 2026. **Revised received:** 12 May 2026. **Accepted:** 12 May 2026
© The Author(s) 2026. Copyright and licence statements to be updated by the publisher during production.

The considered hyperparameter values discussed in the corresponding papers and public code repositories can be found in Table S1. For *Leiden*, we iteratively increase the resolution parameter until the desired number of clusters is obtained. We trained and evaluated methods using GPU when available; otherwise, we utilized CPU.

Table S1 Model hyperparameters.

Model	Hyperparameters
SpaceFlow	spatial_regularization_strength: $[0.1, 0.2, 0.5]$; n_neighbors: $[25, 50]$
BayesSpace	model: [*normal*, t]; gamma: $[1, 2, 3]$
stLearn	weights: [weights_matrix_all, weights_matrix_pd_gd, weights_matrix_pd_md, weights_matrix_gd_md, gene_expression_correlation, physical_distance, morphological_distance]
GraphST	alpha: $[1, 10, 20]$; beta: $[1, 10, 20]$; lambda1: $[1, 10, 20]$; lambda2: $[1, 10, 20]$; radius: $[0, 50]$
STAGATE	k_cutoff: $[6, 9, 12, 15]$; hidden_dims: $[[512, 30], [256, 30], [512, 60], [256, 60]]$; pre_resolution: $[0.2, 0.4, 0.6]$
MUSE	lambda_regul: $[1, 5, 10]$; lambda_super: $[1, 5, 10]$
SpaGCN	s: $[1, 2, 3]$; histology: [*False*, *True*]; n_neighbors: $[5, 15, 30]$
AESTETIK	morphology_weight: $[0, 1, 1.5, 2, 3]$; window_size: $[5, 7]$; refine_cluster: [*False*, *True*]
PearlST	epochs: $[500, 1000]$; hidden2: $[16, 32]$; lr: $[0.001, 0.0005]$

S2. Data

S2.1. LIBD Human DLPFC

The LIBD Human DLPFC dataset (Maynard et al. 2021) comprises 12 tissue slices obtained from the dorsolateral prefrontal cortex (DLPFC) brain region, sequenced using Visium from 10x Genomics™ (Table S2). Each spot is manually annotated based on brain cytoarchitecture and known marker genes.

Table S2 The LIBD Human DLPFC contains 3 patients (A, B, C) with two pairs of spatially adjacent replicates (1, 2) resulting in 12 tissue slices.

SampleID	Replicate Pair	Patient
151507	1	A
151508	1	A
151509	2	A
151510	2	A
151669	1	B
151670	1	B
151671	2	B
151672	2	B
151673	1	C
151674	1	C
151675	2	C
151676	2	C

S2.2. Human Breast Cancer

The Human Breast Cancer dataset (Wu et al. 2021) contains 6 tissue slices sequenced using Visium from 10x Genomics™ in 2 independent biological labs (Table S3). Due to incomplete and low-quality annotation, slice CID4290 is removed from the analysis. Each spot is manually annotated based on Loupe v.4.0.0 software (10x Genomics™).

Table S3 The Human Breast Cancer dataset contains 6 samples sequenced in 2 independent biological labs — A and B.

SampleID	Lab
CID4465	A
CID44971	A
CID4535	A
CID4290	A
1142243F	B
1160920F	B

S2.3. Tumor Profiler

The metastatic melanoma dataset comprises 18 tissue slices sequenced using Visium from 10x Genomics™ from the Tumor Profiler study (Irmisch et al. 2021). The tissue slices originate from 9 tissue regions (6.5×6.5 mm^2) from 7 donors, each characterized by one of the following immune subtypes: immune desert, immune excluded, or inflamed. It contains 1 replicate for each tissue region — resulting in 18 tissue slices. The H&E images have (or are scaled to) a resolution of 0.30μm/pixel with a spot radius of 160 pixels. The data is generated using 10x Genomics™ *Space Ranger v3.0.0*. The ground truth annotations were generated using histopathology software (HALO AI™ (Indica Labs, Corrales, NM, USA)), classifying the spots into one of the following categories: tumor, stroma, normal lymphoid, and blood/necrosis. Following that, the model predictions underwent manual review by a pathologist.

S2.4. CosMx NanoString™ Human Liver

The CosMx NanoString™ Human Liver dataset, derived from normal liver and hepatocellular carcinoma tissues, was produced using the CosMx Human Universal Cell Characterization RNA panel (He et al. 2022). We used the provided raw Morphology2D Normalized TIFF images with 5 channels, corresponding to the expression level of segmentation markers (Table S4), to create an RGB image with 3 channels by applying PCA. The provided cell profiles are grouped into Field of Views (FOVs) of around 1500 cells each along with their spatial coordinates, and cell type (304 FOVs normal, 383 FOVs cancer). The *Hep* cell groups were merged. Cells situated within a 55-pixel distance from the image boundaries are removed. For computational efficiency, we sample 100 FOVs per condition.

Table S4 Channel order and the corresponding segmentation marker in CosMx NanoString™ Human Liver dataset.

Channel	Liver Normal	Liver Cancer
1	PanCK	PanCK
2	CK8/18	CD68
3	CD298/B2M	CD298/B2M
4	CD45	CD45
5	DAPI	DAPI

S2.5. MERFISH Mouse Hypothalamus

The MERFISH mouse hypothalamus dataset (Moffitt et al. 2018) comprises 12 tissue sections from the hypothalamic preoptic region of Animal 1, profiled using MERFISH at single-cell resolution with 155 targeted genes and approximately 5000–6000 cells per section (15 cell type classes). The data was obtained from the Dryad repository (`https://doi.org/10.5061/dryad.8t8s248`) and processed using the BASS analysis pipeline (Li and Song 2022).

S2.6. Simulated data

We adapted the simulation approach suggested in Bao et al. (2022) by introducing spatial structure in the experiment. Briefly, relying on simulated ground truth labels, we simulate transcriptomics and morphology modalities, allowing partial observation of true clusters within each modality individually. However, combining both modalities enables the identification of all clusters. Spatial coordinates are incorporated by sorting the ground truth in spatial space. We generated 3 datasets with 5 samples (2500 cells/sample) and 5, 10, and 15 clusters each. For the runtime analysis, we generated one tissue slice with 10 million spots and 5 clusters.

S2.7. Runtime analysis

We applied AESTETIK with morphology weight 1.5, window size 3 and a batch size 100 000. Due to the large number of spots, we used *K-Means* clustering from the *sklearn* package. The model was trained on NVIDIA™ V100 GPU with 32G RAM. We conducted runtime benchmarking for three key functions of AESTETIK v0.0.1: grid building (`prepare_input_for_model()`), training (`train()`), and inference (`compute_spot_representations()`).

S3. Supplementary figures

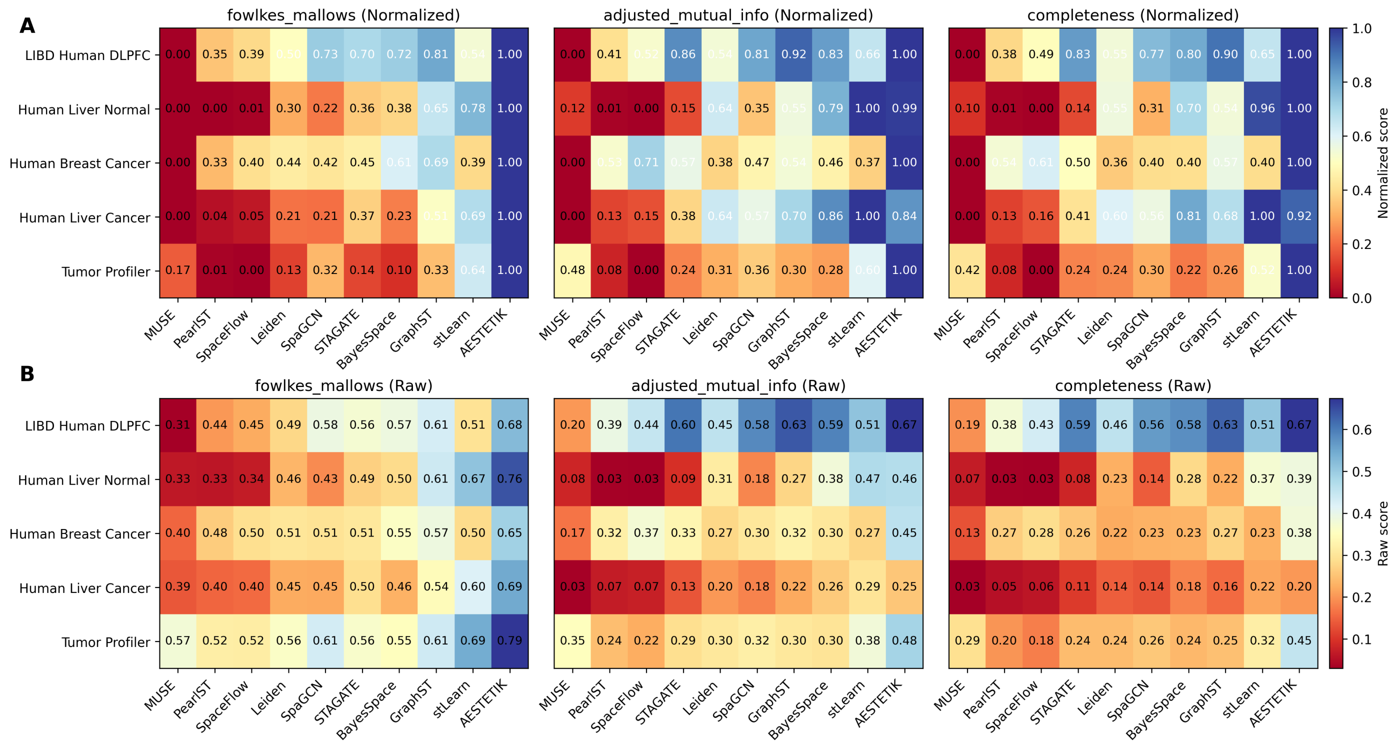

Figure S1 Heatmap comparing spatial transcriptomics integration models across five datasets. Models are evaluated using Fowlkes–Mallows, Adjusted Mutual Information, and Completeness scores. The first row shows row-wise normalized scores, while the second row shows the raw scores.

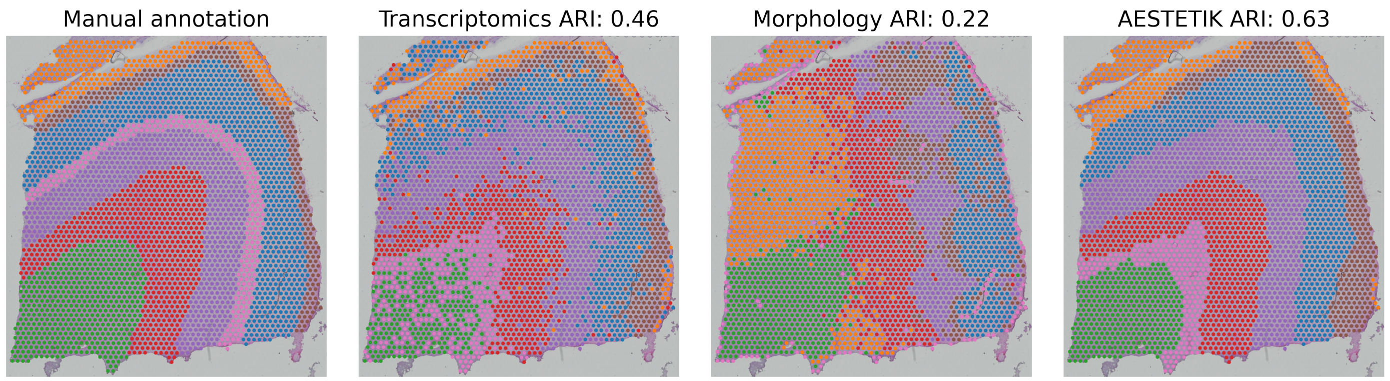

Figure S2 Comparison of cluster assignments for slice 151676 from the LIBD human DLPFC dataset. Transcriptomics only vs. Morphology only vs. AESTETIK.

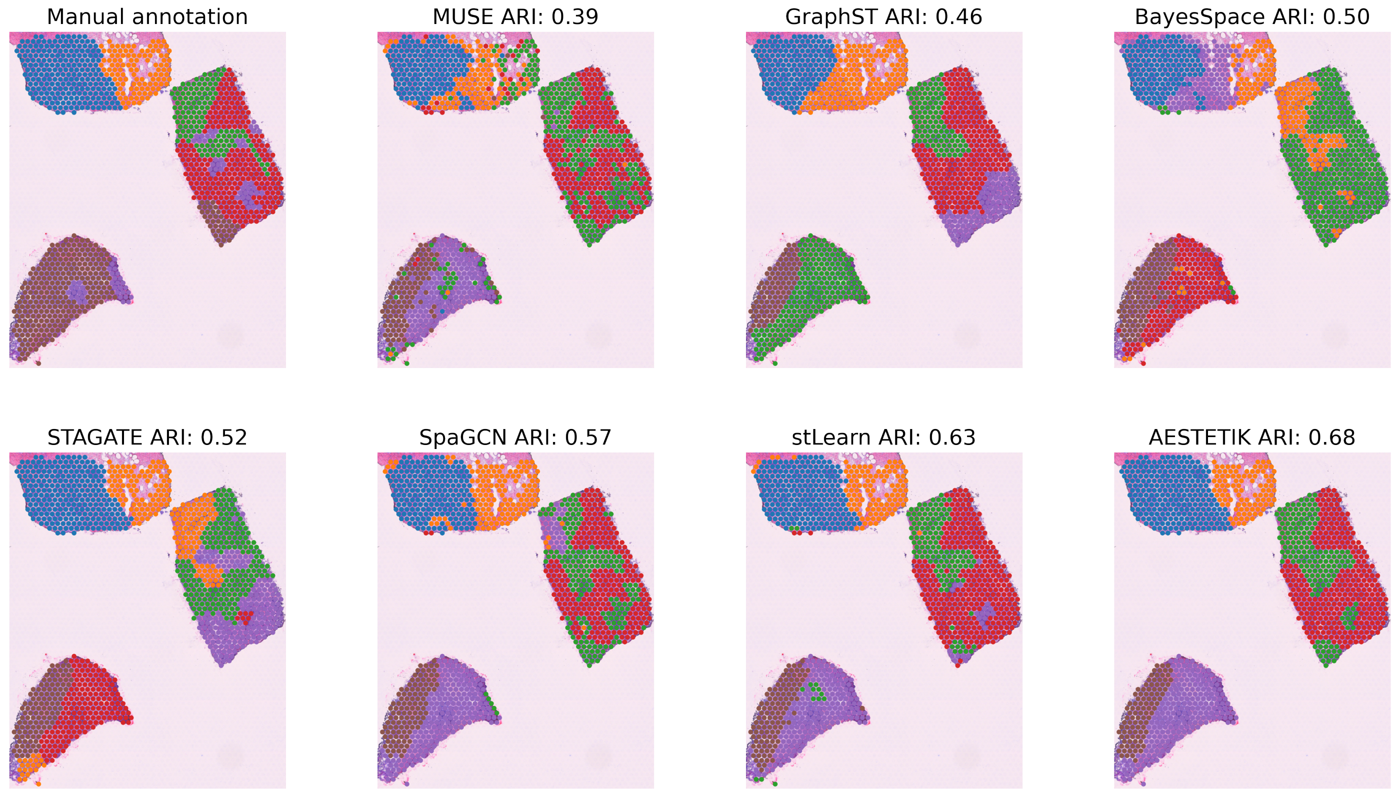

Figure S3 Comparison of cluster assignments for slice CID44971 from the Human Breast Cancer dataset.

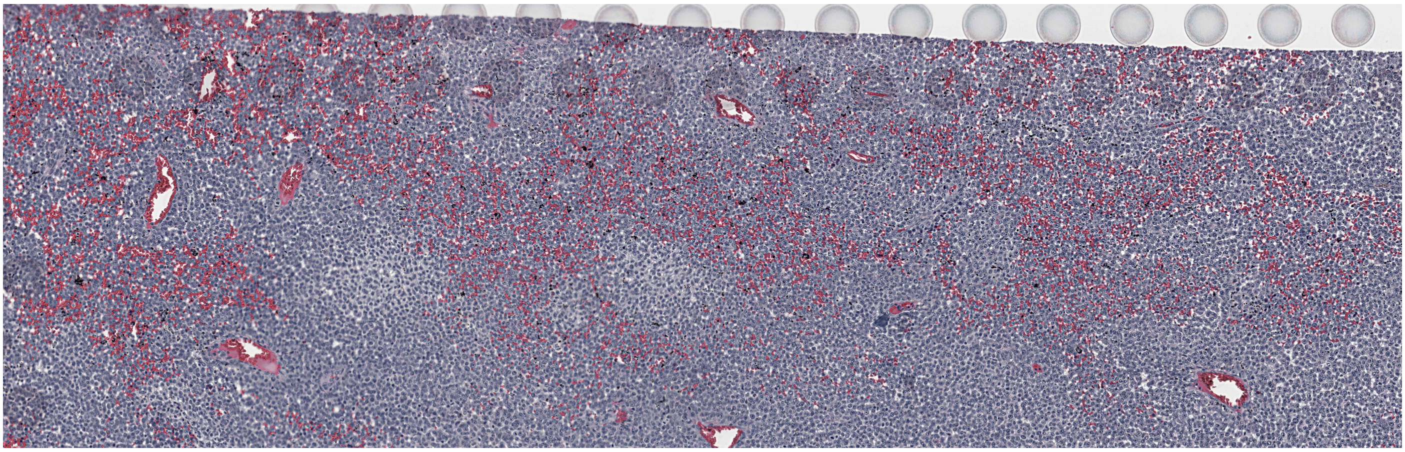

Figure S4 Zoom into the hemorrhage region highlighted in Fig. 3A,B of the main manuscript for slice MACEGEJ-2-2.

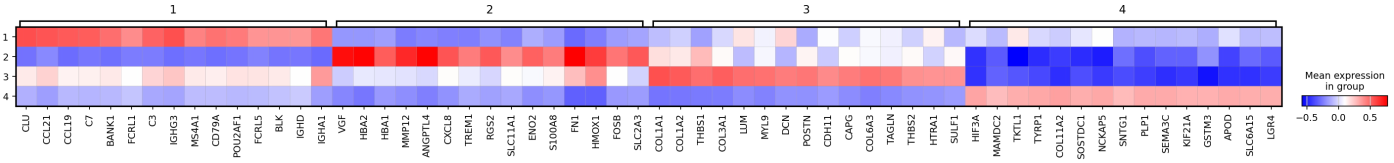

Figure S5 Cluster marker genes for slice MACEGEJ-2-2 from the Tumor Profiler dataset. Cluster 1 corresponds to spots enriched with normal lymphoid cells; cluster 2 — blood and necrosis; cluster 3 — stroma; cluster 4 — tumor.

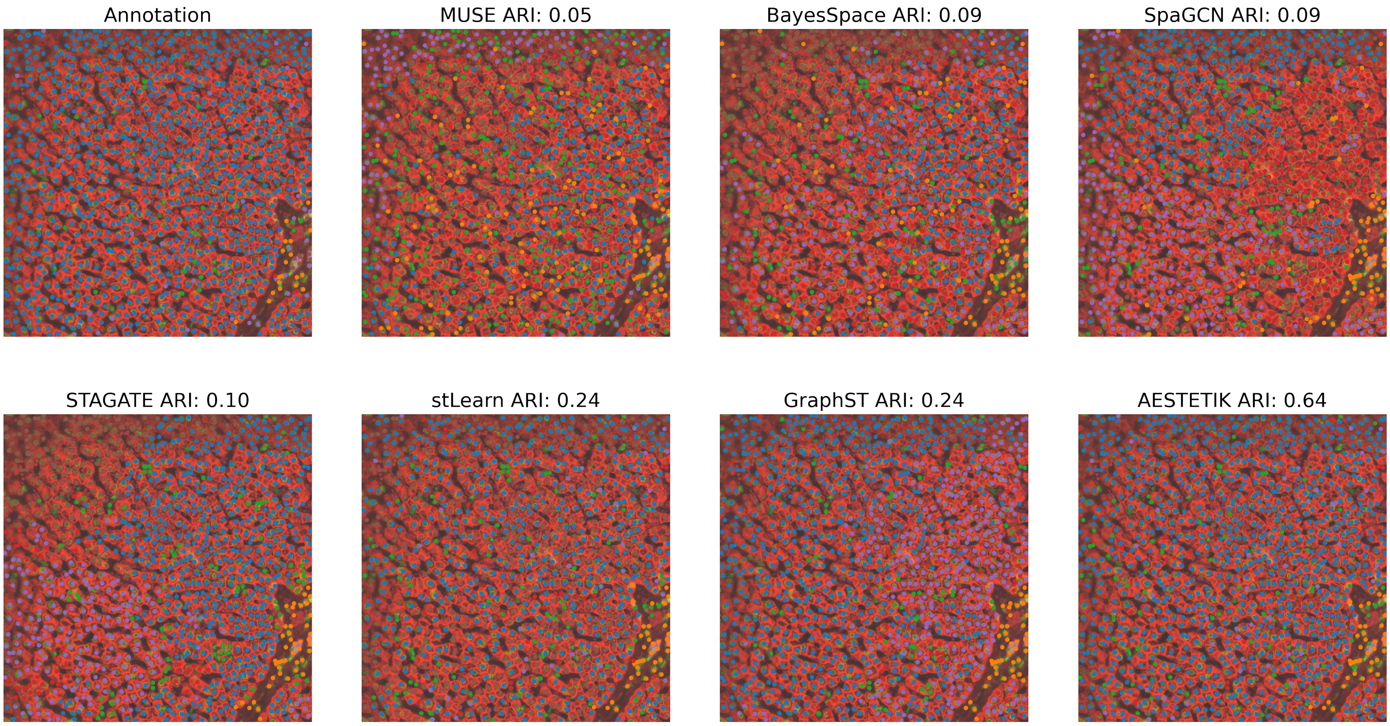

Figure S6 Comparison of cluster assignments for FOV 159 from the CosMx NanoString™ Liver Normal dataset.

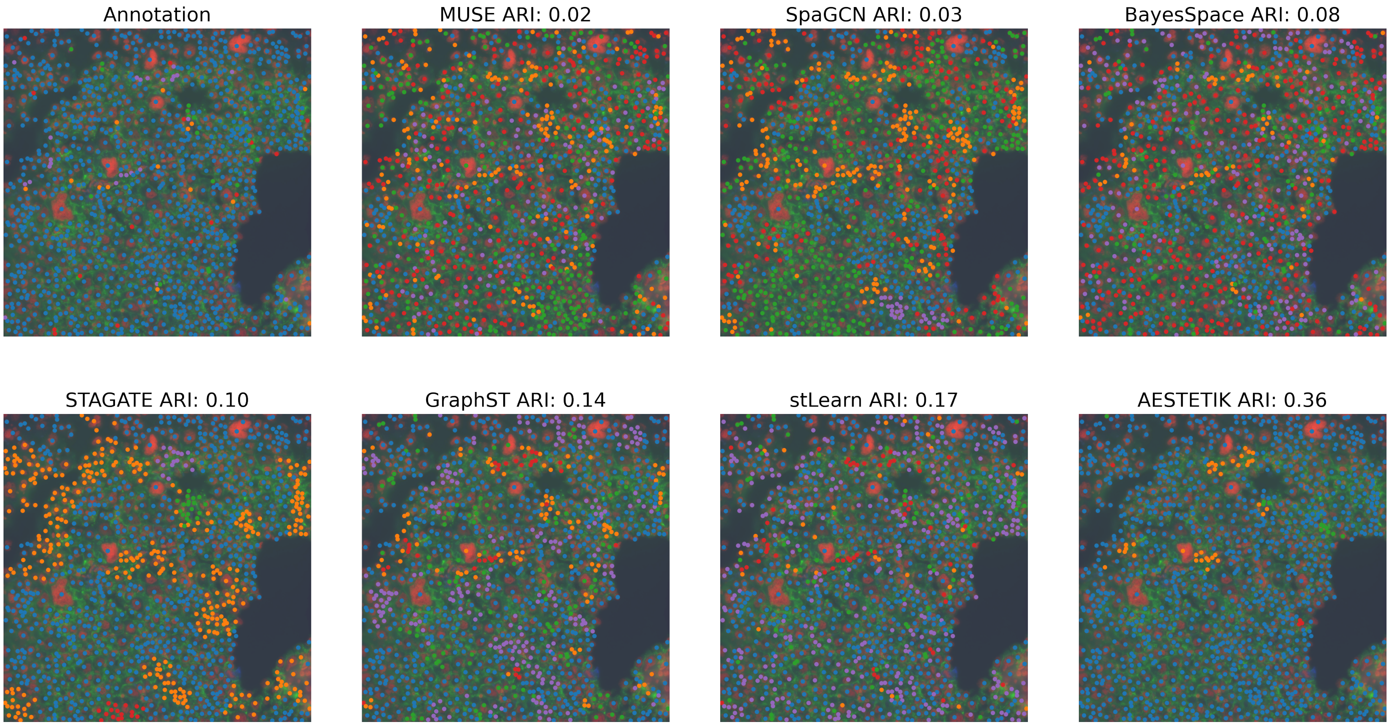

Figure S7 Comparison of cluster assignments for FOV 201 from the CosMx NanoString™ Liver Cancer dataset.

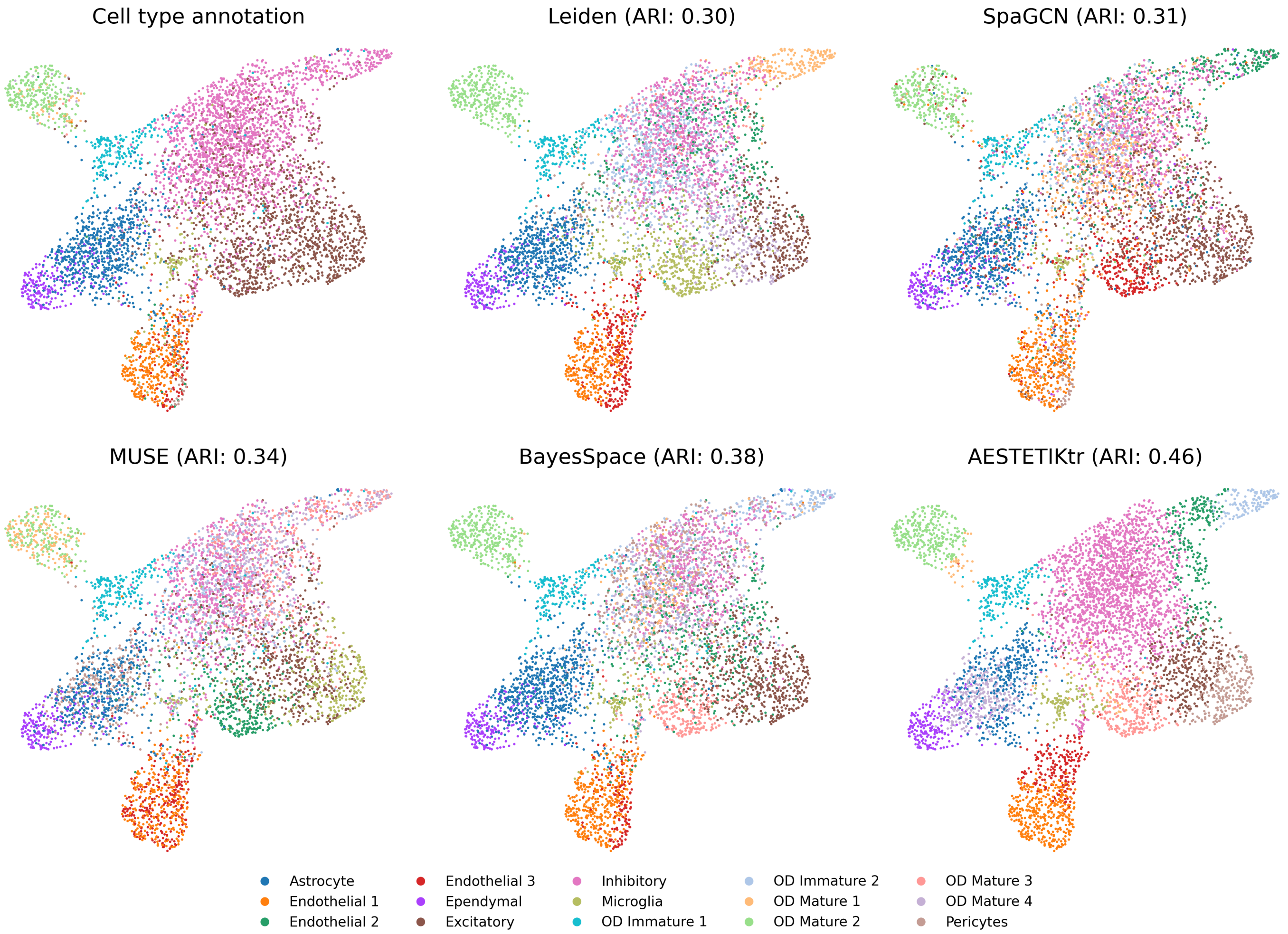

Figure S8 UMAP visualization of cluster assignments for Section 12 from the MERFISH Mouse Hypothalamus dataset. UMAP embeddings computed from the AESTETIK latent space, colored by ground truth cell type annotations and predicted clusters from the top five performing methods. AESTETIK (transcriptomics + spatial only, $\alpha = 0$) achieves an ARI of 0.46 on this image-free spatial transcriptomics platform.

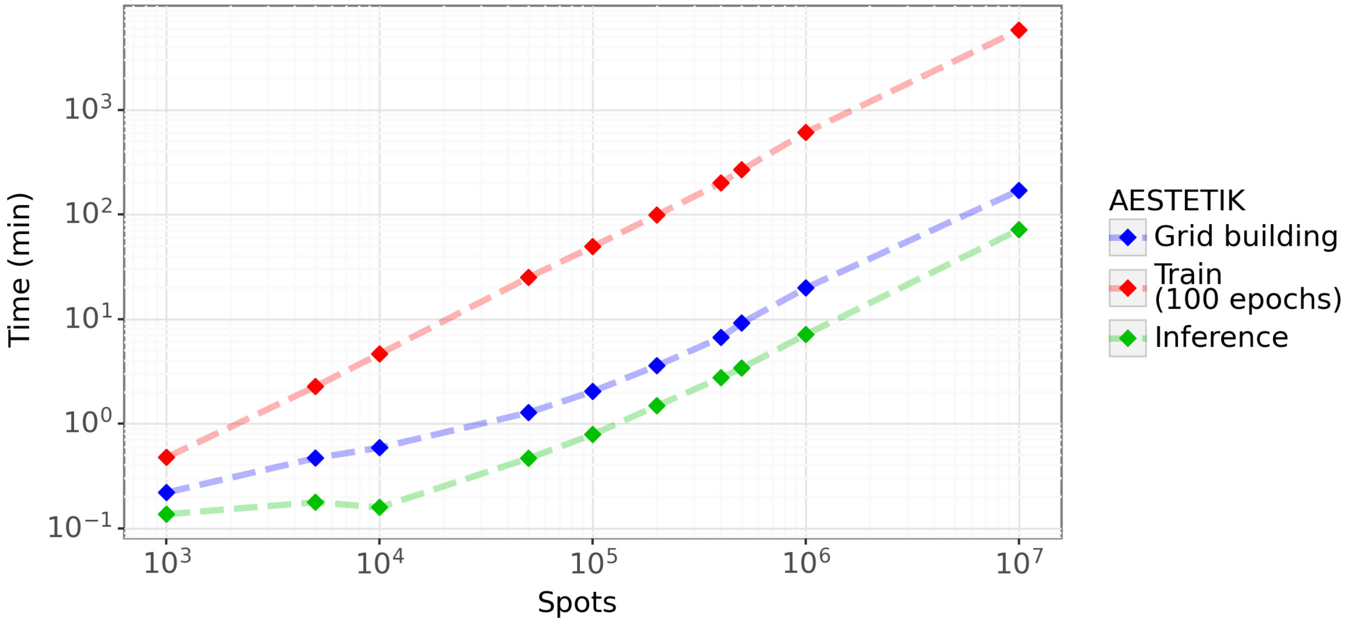

Figure S9 AESTETIK scales to millions of spots. The x-axis represents the number of spots and the y-axis — the time in minutes.

References

F. Bao, Y. Deng, S. Wan, S. Q. Shen, B. Wang, Q. Dai, S. J. Altschuler, and L. F. Wu. Integrative spatial analysis of cell morphologies and transcriptional states with MUSE. *Nature biotechnology*, 40(8):1200–1209, 2022.

K. Dong and S. Zhang. Deciphering spatial domains from spatially resolved transcriptomics with an adaptive graph attention auto-encoder. *Nature communications*, 13(1):1739, 2022.

S. He, R. Bhatt, C. Brown, E. A. Brown, D. L. Buhr, K. Chantranuvatana, P. Danaher, D. Dunaway, R. G. Garrison, G. Geiss, et al. High-plex imaging of RNA and proteins at subcellular resolution in fixed tissue by spatial molecular imaging. *Nature Biotechnology*, 40(12):1794–1806, 2022.

J. Hu, X. Li, K. Coleman, A. Schroeder, N. Ma, D. J. Irwin, E. B. Lee, R. T. Shinohara, and M. Li. SpaGCN: Integrating gene expression, spatial location and histology to identify spatial domains and spatially variable genes by graph convolutional network. *Nature methods*, 18(11):1342–1351, 2021.

A. Irmisch, X. Bonilla, S. Chevrier, K.-V. Lehmann, F. Singer, N. C. Toussaint, C. Esposito, J. Mena, E. S. Milani, R. Casanova, et al. The tumor profiler study: integrated, multi-omic, functional tumor profiling for clinical decision support. *Cancer Cell*, 39(3):288–293, 2021.

Z. Li and X. Song. BASS: multi-scale and multi-sample analysis enables accurate cell type clustering and spatial domain detection in spatial transcriptomic studies. *Genome Biology*, 23(1):56, 2022.

Y. Long, K. S. Ang, M. Li, K. L. K. Chong, R. Sethi, C. Zhong, H. Xu, Z. Ong, K. Sachaphibulkij, A. Chen, et al. Spatially informed clustering, integration, and deconvolution of spatial transcriptomics with GraphST. *Nature Communications*, 14(1):1155, 2023.

K. R. Maynard, L. Collado-Torres, L. M. Weber, C. Uytingco, B. K. Barry, S. R. Williams, J. L. Catallini, M. N. Tran, Z. Besich, M. Tippani, et al. Transcriptome-scale spatial gene expression in the human dorsolateral prefrontal cortex. *Nature neuroscience*, 24 (3):425–436, 2021.

J. R. Moffitt, D. Bambah-Mukku, S. W. Eichhorn, E. Vaughn, K. Shekhar, J. D. Perez, N. D. Rubinstein, J. Hao, A. Regev, C. Dulac, et al. Molecular, spatial, and functional single-cell profiling of the hypothalamic preoptic region. *Science*, 362(6416):eaau5324, 2018.

D. Pham, X. Tan, B. Balderson, J. Xu, L. F. Grice, S. Yoon, E. F. Willis, M. Tran, P. Y. Lam, A. Raghubar, et al. Robust mapping of spatiotemporal trajectories and cell–cell interactions in healthy and diseased tissues. *Nature communications*, 14(1):7739, 2023.

H. Ren, B. L. Walker, Z. Cang, and Q. Nie. Identifying multicellular spatiotemporal organization of cells with SpaceFlow. *Nature communications*, 13(1):4076, 2022.

H. Wang, J. Zhao, Q. Nie, C. Zheng, and X. Sun. Dissecting spatiotemporal structures in spatial transcriptomics via diffusion-based adversarial learning. *Research*, 7:0390, 2024.

S. Z. Wu, G. Al-Eryani, D. L. Roden, S. Junankar, K. Harvey, A. Andersson, A. Thennavan, C. Wang, J. R. Torpy, N. Bartonicek, et al. A single-cell and spatially resolved atlas of human breast cancers. *Nature genetics*, 53(9):1334–1347, 2021.

E. Zhao, M. R. Stone, X. Ren, J. Guenthoer, K. S. Smythe, T. Pulliam, S. R. Williams, C. R. Uytingco, S. E. Taylor, P. Nghiem, et al. Spatial transcriptomics at subspot resolution with BayesSpace. *Nature biotechnology*, 39(11):1375–1384, 2021.
